# Predictors for Returning to Paid Work after Transient Ischemic Attack and Minor Ischemic Stroke

**DOI:** 10.3390/jpm12071109

**Published:** 2022-07-06

**Authors:** Corentin A. Wicht, Camille F. Chavan, Jean-Marie Annoni, Philippe Balmer, Jérôme Aellen, Andrea M. Humm, Fabienne Crettaz von Roten, Lucas Spierer, Friedrich Medlin

**Affiliations:** 1 Neurology Unit, Medicine Section, Faculty of Science and Medicine, University of Fribourg, 1700 Fribourg, Switzerland; corentin.wicht@unifr.ch (C.A.W.); jean-marie.annoni@unifr.ch (J.-M.A.); lucas.spierer@unifr.ch (L.S.); 2 Stroke Unit and Unit of Neurology, Department of Internal Medicine, Cantonal Hospital, 1752 Villars-sur-Glâne, Switzerland; camille.chavan@h-fr.ch (C.F.C.); philippe.balmer@h-fr.ch (P.B.); andrea.humm@h-fr.ch (A.M.H.); 3 Neuropsychology Unit, Cantonal Hospital, 1752 Villars-sur-Glâne, Switzerland; 4 Department of Radiology, Cantonal Hospital, 1752 Villars-sur-Glâne, Switzerland; jerome.aellen@h-fr.ch; 5 Institute of Sport Sciences, University of Lausanne, 1015 Lausanne, Switzerland; fabienne.crettazvonroten@unil.ch

**Keywords:** return to work, minor ischemic stroke, transient ischemic attack, hyperlipidemia

## Abstract

This study aims to determine which factors within the first week after a first-ever transient ischemic attack (TIA) or minor ischemic stroke (MIS) are associated with stroke survivors’ ability to return to either partial or full time paid external work (RTpW). In this single-center prospective cohort study, we recruited 88 patients with first-ever TIA or MIS (NIHSS ≤ 5). Bivariate analyses were conducted between patients that did (RTpW) or did not return to paid work (noRTpW) within 7 days after stroke onset and at 3-months follow-up. Then, we conducted multivariate logistic and negative binomial regression analyses assessing (i) which factors are associated with RTpW at 3 months (ii) the likelihood that patients would RTpW at 3 months and (iii) the number of months necessary to RTpW. Overall, 43.2% of the patients did not RTpW at 3 months. At 3-months follow-up, higher anxiety/depression and fatigue-related disabilities were associated with noRTpW. Multivariate analysis showed that higher NIHSS scores at onset and hyperlipidemia (LDL cholesterol > 2.6 mmol/L or statins at stroke onset) were associated with noRTpW at 3 months. Stroke severity and/or newly diagnosed hypercholesterolemia at stroke onset in TIA or MIS patients were associated with not returning to paid work at 3 months.

## 1. Introduction

Returning to paid work (RTpW) is of utmost importance for stroke survivors and closely linked to a better quality of life [1]. The proportion of stroke survivors RTpW varies from 38% to 55% according to former studies [2,3,4]. RTpW also helps minimize productivity loss of stroke survivors and in turn the socioeconomic impact of stroke in modern countries [5]. In general, stroke severity is considered as the main contributor to RTpW [6] with acute signs of cortical dysfunction such as language impairment or hemispatial neglect being negatively correlated with return to work [7]. Demographic characteristics such as age [8], sex [9], type of work [9] and living arrangements [10] contribute to differences in the tendency to RTW (i.e., return to either paid or unpaid work). Moreover, emotional factors such as psychological well-being [10] as well as lesion size and location [3] have been reported as predictors. Finally previous cardiovascular risk factors such as high blood pressure and hyperlipidemia possibly mediated through increasing stroke severity [11] and post-stroke physical disabilities [12] have been suggested to indirectly impact RTW propensity [13,14].

Yet, these factors were mostly identified in population with severe stroke, leaving unresolved whether the same factors account for RTpW in first-ever transient ischemic attack (TIA) or minor ischemic stroke (MIS), a population representing about 40% of all ischemic strokes [15]. Since in TIA and MIS patients, disabling deficits are often unrecognized due to unmeasured neurological deficits by the National Institutes of Health Stroke Scale (NIHSS) score from the incident stroke, the factors influencing RTpW in this population may differ from those with more severe stroke. Only scarce evidence has identified predictors for RTpW in the TIA and MIS populations. Previous work showed that severe fatigue [16], was related to alteration of professional activity and that post-stroke fatigue was associated with poorer functional outcome [17], while stroke severity (NIHSS score) correlated with increased risks of unemployment. Finally, Carlsson et al. [18] observed in patients not RTpW, that nearly all displayed symptoms of aestheno-emotional disorder. 

The present prospective cohort study aims to determine which factors within the first weeks after a first-ever TIA or MIS are associated with stroke survivors’ ability to RTpW.

## 2. Materials and Methods

### 2.1. Study Population & Materials

Ninety-five patients with acute MIS or TIA (ABCD^2^ score > 3) were recruited between December 2015 and July 2019 in the swiss-certified Stroke Unit of the County Hospital of Fribourg. Prior to inclusion, patients provided written consent. The Ethics Committee for research on humans of the Canton of Vaud (ECCV) approved the protocol (REC Ref: 399/15).

We included men or women aged ≥18 and <65, with (i) full time or part time work prior to study enrolment, (ii) sufficient level in German or French to understand and reply to study questions and with (iii) first-ever TIA (tissue-based definition) or acute minor ischemic stroke (<7 days after stroke onset) defined as NIHSS score ≤ 5 at admission. Exclusion criteria were (i) recurrent stroke or subarachnoid hemorrhage, (ii) severe aphasia (defined as ≥3 on the NIHSS item 9), (iii) relevant neurological, psychiatric, or neuropsychiatric history of comorbidity, (iv) pre-existing cognitive impairment based on the Informant Questionnaire on Cognitive Decline in the Elderly short form (IQ-CODE) and/or history taken from the patient’s relatives and/or knowledgeable informants and (vi) alcohol dependency or other chronic toxic abuse. Patients who met the inclusion/exclusion criteria were clinically evaluated (clinical examination, questionnaires, and neuropsychological assessment) within the first week after the initial event and then at 3 months in the outpatient clinic.

Cognitive decline and dementia were assessed by relative or friends using the IQ-CODE. From the acute phase, demographics (age, sex, type of work, living situation, educational level), clinical (hypertension, smoking, BMI), biological (hyperlipidemia, i.e., defined as statin treatment before stroke onset or LDL-C > 2.6 mmol/L, Thyroid-Stimulating Hormone (TSH)) and radiological variables (lesion site) were collected and analyzed. Stroke pathophysiology was classified according to the Trial of Org 10172 in Acute Stroke Treatment procedure (TOAST) [19] with dissections and multiples causes recorded as additional mechanism. Additionally, self-evaluation with the Hospital Anxiety and Depression Scale (HAD), Fatigue Impact Scale (FIS) and stroke severity by NIHSS-certified personnel were assessed. Functional outcome was assessed with the mRS and cognitive impairment was evaluated using the Montreal Cognitive Assessment (MoCA). All questionnaires were administered during the first clinical evaluation (within 7 days after the initial event) and at 3-months follow-up.

Brain imaging was routinely performed at admission or prior admission to the emergency department/hospital using Magnetic Resonance Imaging (MRI; 3 T or 1.5 T) or if not feasible (e.g., claustrophobic patients) by angio-CT scan.

### 2.2. Data Analysis Section

#### 2.2.1. Outcome Measures

The primary outcome, i.e., RTpW, corresponded to the ability/inability to return to either partial or full time paid external work at 3-months. Patients were divided in two groups (i.e., between-subject factor “Group”) based on the: (i) return to partial/full time paid work (i.e., same working quota as pre-stroke work, i.e., RTpW) or (ii) return to lower working quota as compared to pre-stroke work or no return to work at all (i.e., NoRTpW). Additionally, for the repeated measures, each patient was assessed at two timepoints (i.e., within-subject factor “Time”): (i) within the first week after the initial event (i.e., baseline) and (ii) at 3 months in the outpatient clinic. Another main outcome was the delay in months needed to RTpW only for the patients who did return to work.

#### 2.2.2. Statistics

The alpha level was set at 5% for all statistical analyses. We explored the univariate normality of data distribution relying on the Shapiro-Wilk test and on the skewness and kurtosis acceptable range of ±2 for parametric analyses [20]. Since all our distributions were asymmetrical, we computed (i) Mann-Whitney U tests when contrasting NoRTpW vs. RTpW for baseline measures, and (ii) robust repeated measures ANOVAs (i.e., with 20% trimmed means) when contrasting NoRTpW vs. RTpW groups at baseline Vs at 3 months, using the *WRS2* package [21]. Of note, data averages are reported respectively as median/IQR and as trimmed mean ± SD. Additionally, post-hoc Yuen’s tests were computed and adjusted for multiple comparisons using the False Discovery Rate (FDR) for each dependent variable’s set of contrasts. 

Then, we computed (i) binomial logistic regression and (ii) negative binomial regression models trying to infer, respectively, (i) the probability of patients to be classified in the NoRTpW vs. RTpW groups and (ii) the delay in months needed to RTpW. For each model, we included as regressors the variables showing significant univariate differences when contrasting NoRTpW vs. RTpW groups at baseline (see Supplementary Materials for further details). Finally, we performed a causal mediation analysis using the *Mediation* R package [22] to evaluate the hypothesis that hyperlipidemia’s effect on the likelihood to RTpW might be mediated by stroke severity, indexed by the NIHSS score [11]. We conducted Voxel-Based Lesion-Symptom Mapping (VLSM) analyses with the *NiiStat* toolbox v.1.1 [23] to statistically examine the anatomo-clinical correlation between the presence/absence of a lesion at each specific voxel and the ability/inability to RTpW [24]. Individual patients’ lesions demarcation was performed on each axial slice of Diffusion Weighted Images relying on *Clusterize* v.1.0 beta [25]. Each lesion mask was then normalized to the Montreal Neurological Institute’s brain space using the *Clinical* toolbox v.7/7/2016 [26] and SPM12 (v.7487) in Matlab R2018b (The Mathworks, Inc., Natick, MA, USA). FDR-adjusted Chi-square one-tailed statistics were conducted only testing voxels damaged in at least 5 patients.

The analysis code written in R 4.0.2 is made freely available (https://doi.org/10.5281/zenodo.4551349 (accessed on 19 February 2021)).

## 3. Results

**Figure 1:**
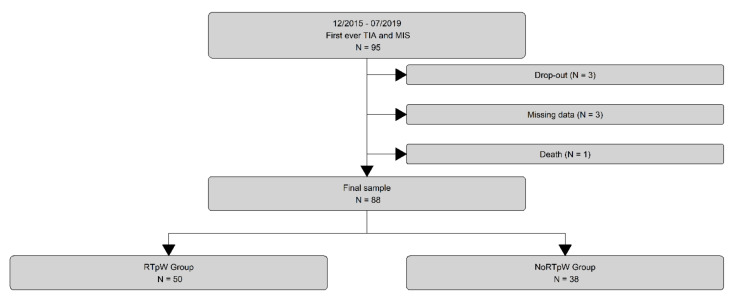
Study flow diagram. MIS = Minor ischemic stroke; NoRTpW = No Return to Paid Work; RTpW = Return to Paid Work; TIA = Transient ischemic attack.

Among the 95 recruited patients, we excluded 7 of them from the analyses (see for details Figure 1). The final cohort included 88 patients of which 29.5% were women with a mean age of 51.6 (±10 years). The most relevant demographic, clinical and biological characteristics dichotomized for employment status at 3 months are reported in Table 1 (for a complete version see Supplementary Table S31 of the online only data supplement). Globally, 56.8% of our patients returned to paid work at 3 months with a mean delay of 2 months (±2.45; Supplementary Figure S28). There was only one patient in the RTpW group who had a recurrent stroke during follow-up.

Univariate statistics (see Table 1 and Supplementary Tables S3–S31) indicated that the patients diagnosed with hyperlipidemia were less likely to returning to paid work (i.e., RTpW group) compared to those that were not (i.e., NoRTpW group; *p* < 0.01). Additionally, stroke severity (NIHSS) scores were lower in the RTpW group (*p* < 0.05), independent of time. At 3 months, RTpW patients, were less anxious and depressed (HAD; *p* < 0.01), reported less cognitive, physical, and psychosocial fatigue (respectively *p* < 0.001, *p* < 0.01, *p* < 0.001) whereas we found no evidence for a difference in MoCA scores between groups and/or time. Regarding other demographics (type of work, living situation), cardiovascular risk factors (hypertension, smoking, BMI), clinical and biological characteristics (lesion site, event type, discharge destination, stroke mechanism, TSH or LDL-C) there was no evidence for a difference between groups. 

**Figure 2:**
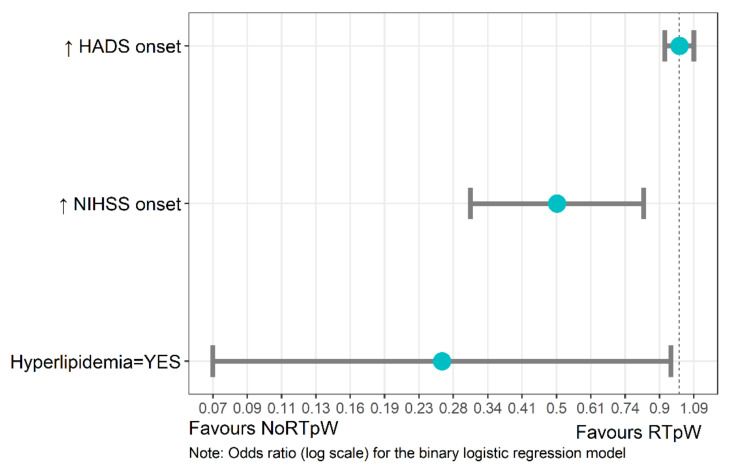
Odds ratio related to the binary logistic regression model. The “↑” symbol represents high scores such that, for e.g., high NIHSS scores at onset will favour NoRTpW. The blue circle represents the odds ratio and the grey bars the 95% confidence intervals.

According to the binomial logistic regression model, only hyperlipidemia and higher NIHSS scores at onset were associated with noRTpW at three months. Patients with hyperlipidemia were nearly four times and patients with higher NIHSS score at baseline two times less likely to RTpW (Figure 2 and Table 2). The full model including all three regressors was statistically significant, *(*χ^2^(3) = 13.25, *p* < 0.01), indicating that the model could accurately distinguish between RTpW vs. NoRTpW patients with a 68% classification accuracy.

We further conducted a negative binomial regression model to identify factors associated with the delay in time (number of months) to RTpW (Table 3 and Supplementary Figure S28). The model estimating the effect of hyperlipidemia, NIHSS and HAD scores at onset was significant (χ^2^(18) = 56.70, *p* < 0.001) and showed that the estimated number of months needed to RTpW in non-hyperlipidemic patients was overall 1.2 months compared to roughly 2.5 months in hyperlipidemic patients (*p* < 0.05).

**Figure 3:**
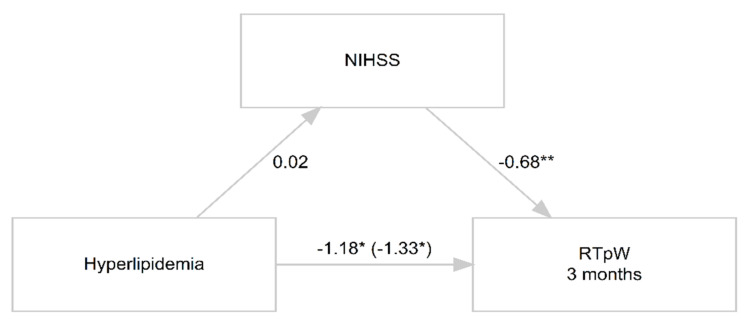
Diagram of three-variable causal mediation analysis results. *p* < 0.001; * *p* < 0.05, ** *p* < 0.01.

In the causal mediation analysis, we could not find support for a mediation of NIHSS on the effect of hyperlipidemia on the likelihood to RTpW. As illustrated in Figure 3, the regression coefficient between hyperlipidemia and NIHSS was non-significant (*p* = 0.94), while the one between NIHSS and RTpW was significant (*p* = 0.006). 

**Figure 4:**
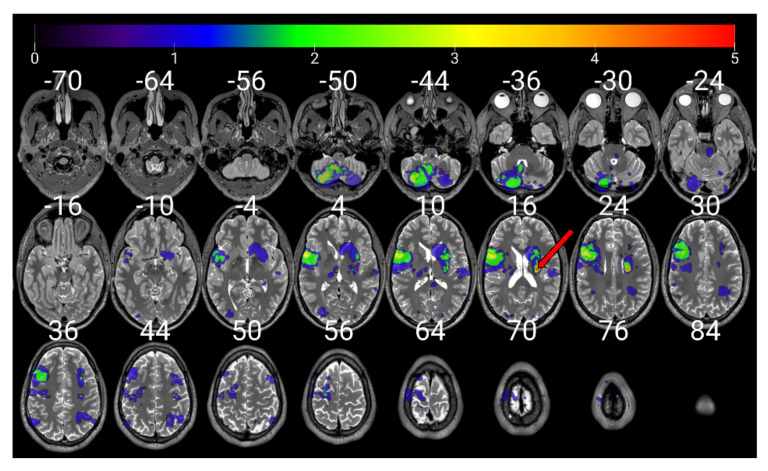
Superposition of the individual MRI lesion masks. Mosaic display of axial slices presented in neurological convention (left hemisphere on the left). The red arrow is pointing at the only cluster where at least 5 patient’s lesions overlapped. The color bar symbolizes the number of patients’ lesions overlapping from 0 to 5.

The VLSM results revealed no evidence for an effect of brain lesion location on RTpW (*p* > 0.05). Yet, lesion overlap map indicated that due to the test being restricted to areas lesioned in at least 5 patients, the statistical analyses of lesion-symptom mapping were conducted in a very small right-hemisphere cluster including the caudate, putamen, thalamus, pallidum, and parts of the cortico-spinal and the corpus callosum white matter tracts (Figure 4). We can thus not exclude that lesion on brain areas outside this cluster may influence RTpW.

## 4. Discussion

In this single-center prospective cohort study, we could demonstrate that 43% of the included 88 TIA or MIS patients, did not RTpW at 3 months after the index cerebrovascular event. Interestingly, RTpW at three months was associated with lower stroke severity measured by the NIHSS score and absence of known or newly diagnosed hyperlipidemia at stroke onset. Inversely, in this study, patients with hyperlipidemia at onset (defined as LDL-C > 2.6 mmol/L or statins at onset) were almost 4 times less likely to RTpW at 3 months and it would require them about 1.5 additional month to RTpW compared to patients without hyperlipidemia. Of note, we found no statistical difference between the RTpW and NoRTpW groups concerning LDL-C and total cholesterol levels at onset or related to the presence or absence of lipid lowering treatment before stroke onset. While the percentage of patients not RTpW is in accordance with previous investigations including stroke patients [3,27], our data confirm that TIA or MIS might have serious long-term consequences in daily life situations.

The present study provides, for the first time, evidence regarding factors at stroke onset associated with RTpW in a population of first-ever TIA and MIS patients. While limited evidence [16,17,18] demonstrated that fatigue, anxiety, cognitive deficits, emotional instability, and stroke severity at onset (i.e., indexed with the NIHSS) are correlated to patients’ ability to RTW, none of the above could draw causal relationships in a prospective study framework. While we partially reproduce previous findings such that NoRTpW patients display higher levels of anxiety, depression and fatigue related disabilities compared to RTW patients, of the aforementioned factors only stroke severity at admission was significantly associated in our sample with the odds of returning or not to paid work.

Most interestingly and to our knowledge not yet reported, we observed a significant association between hyperlipidemia and post-stroke ability to noRTpW. Hyperlipidemia is well known to promote atherosclerosis and stroke occurrence [28]. Approximately half of ischemic stroke patients are diagnosed with hyperlipidemia [29] (~70% in our sample), which makes it a predominant cause of stroke occurrence [30] while its consequences regarding post-stroke recovery remain largely unexplored. Accordingly, Sim et al. [12] illustrated in a sample of hemiparetic stroke patients that a history of hyperlipidemia was related with impairments in physical functioning while Xu et al. [11] showed that higher serum levels of triglycerides, low- and high-density lipoprotein cholesterol levels lead to increased NIHSS and death rate at discharge. More specifically, Zeljkovic et al. [31] reported that solely LDL-C (and not HDL-C) was related to the occurrence of acute ischemic stroke and further in-hospital mortality rate. Again, one obvious explanation is that hypercholesterolemia is a well-known cardiovascular risk factor and somehow a surrogate marker of general vascular disease. Therefore, these patients are more vulnerable for other known vascular complications as well as stroke severity and recurrence with negative impact of working status. In contrast to prior evidence [11], and according to our causal mediation analysis, stroke severity did not mediate the relationship between hyperlipidemia and the likelihood to RTpW in our cohort.

The strengths of our study are its prospective framework, the presence of homogeneous samples across groups and the use of advanced statistical methods while the main limitations are its nonrandomized and monocentric nature as well as the limited sample size, which might alter generalizability of our findings. First, since we only recruited hospitalized patients, selection bias cannot be systematically ruled out, especially for TIA patients, but the latter were less numerous in our cohort (i.e., 4 in each group). Secondly, the size of the sample included in each regression model may have been too small to reach a consistent power [32]. Lastly, our result regarding the negative association between hyperlipidemia and RTpW could be challenged such that the increase in the difficulty to RTpW might be a consequence of the prescription of statins at hospital discharge (e.g., side-effects). While we have demonstrated that there was no evidence for groups differences in term of percentage of patients who were prescribed statins at discharge (Supplementary Table S13 and Figure S13), we could not provide data regarding the type of statins prescribed and their associated side-effects. 

In regard to our findings on hyperlipidemia, Amarenco et al. [33], demonstrated that administration of lipid-lowering drugs after stroke enhanced the neurological recovery of patients. In the same vein, Lakhan et al. [34] showed that patients on statins medication prior stroke occurrence had better neurological outcomes and less serious structural injury, while Moonis et al. [35] suggest that pre- and post-stroke statin use may both enhance stroke recovery. Thus, future studies should investigate how neuro-restoration interventions aiming to enhance brain remodeling following the acute phase of stroke may promote RTpW [36].

Finally, to our knowledge, VLSM analyses have never been applied to MIS populations in relationship to their tendency to RTpW. Putative explanations for the absence of an anatomo-clinical correlation effect are that (i) we could not have MRI confirmation of strokes occurrence in ~16% of patients and (ii) the cohort was composed of patients with acute MIS whose overlapped brain lesions resulted in a poor coverage of the brain (Figure 3). We suggest that this analysis should be repeated in further studies on a larger cohort.

## 5. Conclusions

In conclusion, in our prospective cohort study we showed that in TIA and MIS patients, stroke severity as well as known or newly diagnosed hypercholesterolemia in secondary prevention at stroke onset are the major contributors not to RTpW at three months.

## Figures and Tables

**Table 1:** Demographics and clinical characteristics of the study population according to returning to paid work at three months.

	Overall	NoRTpW	RTpW	Statistics
Demographics				
Gender				*p* = 0.55, φ = 0.06
Male	62	25	37	
Female	26	13	13	
Age	54.00/10.50	53.00/9	54.00/14	*p* = 0.64, r = 0.05
Cardiovascular risk factors and comorbidities				
Hyperlipidemia				*p* = 0.01, φ = 0.29
No	24	5	19	
Yes	58	32	26	
LDL Cholesterol	3.56 ± 0.99	3.60 ± 0.97	3.53 ± 1.02	*p* = 0.76, g = 0.07
TSH	2.07/1.67	2.06/1.31	2.14/1.75	*p* = 0.93, r = 0.01
Cerebrovascular event				
NIHSS onset	0.57 ± 0.71	1.10 ± 1.00	0.32 ± 0.48	B: *p* = 0.04
NIHSS 3m	0.00 ± 0.00	0.21 ± 0.42	0.00 ± 0.00	W: *p* = 0.00
				I: *p* = 0.16
MoCA onset	26.67 ± 1.58	26.43 ± 1.53	26.84 ± 1.70	B: *p* = 0.37
MoCA 3m	26.26 ± 1.21	26.05 ± 1.29	26.44 ± 1.28	W: *p* = 0.16
				I: *p* = 0.62
HAD onset	10.06 ± 3.03	10.32 ± 3.54	9.89 ± 2.71	B: *p* = 0.01
HAD 3m	10.65 ± 3.94	14.27 ± 4.18	7.52 ± 2.86	W: *p* = 0.14
				I: *p* = 0.00
FIS Cognitive onset	26.36 ± 13.22	29.75 ± 15.97	24.17 ± 11.63	B: *p* = 0.00
FIS Cognitive 3m	42.43 ± 19.04	65.24 ± 15.93	24.89 ± 12.64	W: *p* = 0.00
				I: *p* = 0.00
FIS Physical onset	30.74 ± 13.25	35.25 ± 17.84	28.02 ± 10.05	B: *p* = 0.00
FIS Physical 3m	46.86 ± 15.09	60.53 ± 8.24	31.36 ± 13.40	W: *p* = 0.01
				I: *p* = 0.03
FIS Psychosocial onset	27.41 ± 9.99	31.06 ± 11.64	25.05 ± 9.76	B: *p* = 0.00
FIS Psychosocial 3m	40.28 ± 16.73	58.03 ± 9.66	23.07 ± 12.33	W: *p* = 0.00
				I: *p* = 0.00

Note: The columns represent (i) count data for Chi-square statistics (*Χ^2^*), (ii) Median/IQR for Wilcox signed-rank tests (W_s_), (iii) trimmed mean ± SD for robust repeated measures ANOVAs (F), (iv) mean ± SD for independent-samples *t*-tests (t)), and their associated statistical tests and effect sizes. B = Main effect of Between-subject factor (i.e., RTpW vs. NoRTpW); FIS = Fatigue impact scale; HAD = Hospital anxiety and depression scale; I = Interaction effect; MoCA = Montreal cognitive assessment; NIHSS = National institutes of health stroke scale; NoRTpW = No return to paid work group; Pre = Before stroke occurrence; RTpW = Return to paid work group; TSH = Thyroid-stimulating hormone; W = Main effect of Within-subject factor (i.e., onset vs. 3 m).

**Table 2:** Factors associated with returning or not to paid work at 3 months.

	B	SE	Z Value	*p*-Value	Odds Ratios [95% CI]
Constant	1.83	0.83	2.19	0.03 *	6.22 [1.21–31.97]
Hyperlipidemia (YES)	−1.33	0.66	−2.03	0.04 *	0.27 [0.07–0.95]
NIHSS onset	−0.69	0.25	−2.76	0.01 **	0.50 [0.31–0.82]
HAD onset	−0.00	0.04	0.03	0.97	1.0 [0.92–1.09]

Note: R^2^ = 0.13 (Hosmer-Lemeshow), 0.17 (Cox-Snell) and 0.22 (Nagelkerke). Model χ^2^(3) = 13.25, *p* < 0.001; * *p* < 0.05, ** *p* < 0.01.

**Table 3:** Factors associated with the delay in months needed to RTpW.

	Estimate	SE	Z Value	*p*-Value	95% CI	
					Lower	Upper
Constant	−0.46	0.45	−1.02	0.31	−1.43	0.46
Hyperlipidemia (YES)	0.74	0.35	2.09	0.04 *	0.02	1.48
NIHSS onset	0.22	0.12	1.83	0.07	−0.02	0.48
HAD onset	0.04	0.02	1.95	0.05	0.00	0.08

Note: θ = 1.47; R^2^ = 0.20 (McFadden), 0.62 (Cox-Snell) and 0.62 (Nagelkerke). Model χ^2^(18) = 56.70, *p* < 0.001; * *p* < 0.05.

## Data Availability

The data presented in this study are available on reasonable request from the corresponding author. The data are not publicly available due to restrictions on privacy.

## Supplementary Materials

The following supporting information can be downloaded at: https://www.mdpi.com/article/10.3390/jpm12071109/s1, Table S1 Normality Test (shapiro-Wilk); BIVARIATE TESTS: Table S2 Mean age and SD, Tables S3, S4, S7, S12 and S13 Fisher’s Exact Test for Count Data, Tables S5, S6, S8–S11 and S14 Pearson’s Chi-squared test with Yates’ continuity correction, Tables S15–S18 Wilcoxon rank sum test with continuity correction, Tables S19 and S20 Welch Two Sample *t*-test; ROBUST ANOVAs: Tables S21–S23, S25, S27 and S29 ROBUST ANOVA using trimmed means, Tables S24, S26, S28, S30 Yuen’s test using trimmed means, Table S31 Extended Table 1 from manuscript; Binomial Logistic Regression Model: Table S32 Coefficients, Table S33 Deviance residuals, Table S34 Model Fit Measures, Table S35 Pseudo 𝑅^2^ for logistic regression, Table S36 Odds Ratio, Table S37 Residuals statistics, Table S38 Assumption of linearity of the logit (for continuous predictors), Table S39 Durbin-Watson test (Assumption of independence), Table S40 Colinearity Diagnostics, Table S41 Classification Table, Table S42 Predictive Measures; Negative Binomial Regression Model: Table S43 Mean and SD for the RTpWMonths DV, Table S44 Coefficients, Table S45 Pseudo 𝑅2 values, Table S46 Omnibus test, Table S47 Incidence rate ratios, Table S48 MODEL ASSUMPTION: Likelihood ratio test Negative Binomial Reg > Poisson Reg models, Table S49 Residuals statistics, Table S50 Durbin-Watson test (Assumption of independence), Table S51 Colinearity Diagnostics, Table S52 Breusch-Pagan test for heteroskedasticity, Table S53 Predicted probabilities; Causal Mediation model: Table S54 Total effect (IV –> DV), Table 255 Mediator effect (IV –> mediator), Table S56 Direct effect (IV + mediator –> DV), Table S57 Mediation analysis, Table S58 Sensitivity analysis, Figure S1 Descriptives RTpW & Gender percentage, Figure S2 Work change in percentage, Figure S3 Discharge destination, Figure S4 Etiology TOAST, Figure S5 Gender, Figure S6 Type Of Work, Figure S7 Event Type, Figure S8 Living Situation, Figure S9 Smoking, Figure S10 Arterial Hypertension, Figure S11 Hyperlipidemia, Figure S12 Antilipid before stroke, Figure S13 Antilipid at discharge, Figure S14 Lesion Site, Figure S15 Thyroid-Stimulating Hormone, Figure S16 Duration hospitalization, Figure S17 Age, Figure S18 BMI, Figure S19 LDL-Cholesterol, Figure S20 Total Cholesterol, Figure S21 NIHSS, Figure S22 Montreal Cognitive Assessment (MoCA), Figure S23 Hospital Anxiety and Depression scale (HAD), Figure S24 Fatigue Impact Scale (FIS) Cognitive, Figure S25 FIS Physical, Figure S26 FIS Psychosocial, Figure S27 ROC Curve, Figure S28 Number of months needed to RTpW, Figure S29 Assumption of heteroscedasticity, Figure S30 Sensitivity analysis.
