# Supplementary material for: Predictors for Returning to Paid Work after Transient Ischemic Attack and Minor Ischemic Stroke
Source: J Pers Med. 2022 Jul 6;12(7):1109. doi: 10.3390/jpm12071109 (PMC9325246; doi:10.3390/jpm12071109)

SUPPLEMENTAL INFORMATION : Predictors for Returning to Paid Work After Transient Ischemic Attack and Minor Ischemic Stroke

Corentin A. Wicht, MSc   Camille F. Chavan, PhD   Jean-Marie Annoni, MD
Philippe Balmer, MD   Jerome Aellen, MD   Andrea M. Humm, MD
Fabienne Crettaz von Roten, PhD   Lucas Spierer, PhD   Friedrich Medlin, MD

06/07/22

Contents

1 NORMALITY TESTS **3**

2 BIVARIATE TESTS **5**

2.1 RTW and Gender distributions . . . 5
2.2 Work change from 3m to 12m follow-ups . . . 6
2.3 Discharge Destination . . . 7
2.4 Etiology TOAST . . . 8
2.5 Gender . . . 9
2.6 Type of Work . . . 10
2.7 Event Type . . . 11
2.8 Living Situation . . . 12
2.9 Smoking . . . 13
2.10 Arterial Hypertension (HTA) . . . 14
2.11 Hyperlipidemia . . . 15
2.12 Antilipid before Stroke . . . 16
2.13 Antilipid at Discharge . . . 17
2.14 Lesion Site . . . 18

2.15 Thyroid-Stimulating Hormone (TSH) . . . 19
2.16 Duration of Hospitalization . . . 20
2.17 Age . . . 21
2.18 BMI . . . 22
2.19 LDL-Cholesterol (LDL-C) . . . 23
2.20 Total Cholesterol . . . 24

3 ROBUST ANOVAs **25**
3.1 NIHSS . . . 25
3.2 Montreal Cognitive Assessment (MoCA) . . . 26
3.3 Hospital Anxiety and Depression scale (HAD) . . . 27
3.4 Fatigue Impact Scale (FIS) Cognitive . . . 28
3.5 FIS Physical . . . 29
3.6 FIS Psychosocial . . . 30

4 DESCRIPTIVES AND STATISTICAL TESTS SUMMARY (extended Table 1 from manuscript) **31**

5 REGRESSION MODELS **34**
5.1 Binomial Logistic Regression Model - RTpW3m_Percent (Yes/No) . . . 34
5.2 Negative Binomial Regression Model - RTpWMonths . . . 38

6 CAUSAL MEDIATION MODEL **43**

This is the supplemental information for the study:

Predictors for Returning to Paid Work After Transient Ischemic Attack and Minor Ischemic Stroke

Corentin A. Wicht[1]*, MSc; Camille F. Chavan[2], PhD; Jean-Marie Annoni[1,2], MD; Philippe Balmer[2], MD; Jerome Aellen[3], MD; Andrea M. Humm[2], MD; Fabienne Crettaz von Roten[4], PhD; Lucas Spierer[1], PhD and Friedrich Medlin[2], MD

[1]Neurology Unit, Medicine Section, Faculty of Science and Medicine, University of Fribourg, Fribourg, Switzerland;
[2]Stroke Unit and Unit of Neurology, Department of Internal Medicine, Cantonal Hospital, Fribourg, Switzerland;
[3]Department of Radiology, Cantonal Hospital, Fribourg, Switzerland
[4]Lausanne Sports Sciences Institute, University of Lausanne, Lausanne, Switzerland.

Address correspondence and reprint requests to:
Friedrich Medlin, MD
Stroke Unit and Neurology Unit, HFR Fribourg
Chemin des Pensionnats 2, 1708 Fribourg
+41 26 306 22 37 (phone), +41 26 306 22 31 (fax)
Friedrich.Medlin@h-fr.ch

1 NORMALITY TESTS

Table S1: Normality test (shapiro-Wilk)

variable	RTpW_3m_Percent	statistic	p_value	sample	Skewness	Kurtosis
Age	NoRTpW	0.971	0.429	38	-0.435	0.948
Age	RTpW	0.945	0.021	50	-0.357	0.111
BMI	NoRTpW	0.935	0.028	38	1.101	2.624
BMI	RTpW	0.859	0.000	50	1.611	2.938
Duration_hospital	NoRTpW	0.910	0.008	34	1.242	2.731
Duration_hospital	RTpW	0.968	0.313	40	-0.274	-0.423
NIHSS_onset	NoRTpW	0.835	0.000	33	0.494	-1.048
NIHSS_onset	RTpW	0.678	0.000	44	1.828	3.176
TSH	NoRTpW	0.890	0.039	18	1.270	1.739
TSH	RTpW	0.962	0.496	23	0.247	-0.772
MoCA_onset	NoRTpW	0.915	0.008	37	-0.982	0.852
MoCA_onset	RTpW	0.858	0.000	49	-1.421	2.126
MoCA_3m	NoRTpW	0.882	0.002	34	-1.211	1.199
MoCA_3m	RTpW	0.868	0.000	43	-1.603	4.185
HAD_A_onset	NoRTpW	0.967	0.323	37	0.508	-0.240
HAD_A_onset	RTpW	0.964	0.158	46	0.453	0.223
HAD_A_3m	NoRTpW	0.961	0.263	34	0.342	-0.542
HAD_A_3m	RTpW	0.960	0.207	37	0.468	-0.373
HAD_D_onset	NoRTpW	0.849	0.000	37	1.472	1.984
HAD_D_onset	RTpW	0.783	0.000	46	1.889	3.850
HAD_D_3m	NoRTpW	0.925	0.023	34	0.922	0.370
HAD_D_3m	RTpW	0.817	0.000	37	1.512	1.795
HAD_TOTAL_onset	NoRTpW	0.960	0.216	36	0.592	-0.019
HAD_TOTAL_onset	RTpW	0.897	0.001	46	1.409	2.982
HAD_TOTAL_3m	NoRTpW	0.976	0.659	34	0.158	-0.627
HAD_TOTAL_3m	RTpW	0.915	0.008	37	1.005	0.531
FIS_C_onset	NoRTpW	0.911	0.012	32	0.758	-0.195
FIS_C_onset	RTpW	0.911	0.004	40	0.985	0.894
FIS_C_3m	NoRTpW	0.912	0.013	32	-0.733	-0.275
FIS_C_3m	RTpW	0.918	0.011	36	0.774	0.005
FIS_P_onset	NoRTpW	0.933	0.047	32	0.133	-1.295
FIS_P_onset	RTpW	0.935	0.024	40	0.821	0.893
FIS_P_3m	NoRTpW	0.914	0.015	32	-0.992	1.354
FIS_P_3m	RTpW	0.934	0.034	36	0.670	-0.264
FIS_S_onset	NoRTpW	0.961	0.290	32	0.437	-0.505
FIS_S_onset	RTpW	0.942	0.041	40	0.830	0.757
FIS_S_3m	NoRTpW	0.931	0.042	32	-0.949	0.765
FIS_S_3m	RTpW	0.914	0.008	36	0.846	0.052
RTpWMonths	NoRTpW	0.727	0.000	27	2.501	7.606
RTpWMonths	RTpW	0.527	0.000	50	4.609	27.025
Cholesterol_ldl	NoRTpW	0.984	0.850	37	0.001	-0.394
Cholesterol_ldl	RTpW	0.957	0.081	47	0.807	1.949
Cholesterol_total	NoRTpW	0.979	0.685	37	-0.066	-0.358

Cholesterol_total	RTpW	0.968	0.226	46	0.419	1.812

2 BIVARIATE TESTS

2.1 RTW and Gender distributions

Table S2: Mean age and SD

Mean	SD
51.568	9.997

Figure S1: Descriptives RTpW & Gender percentage

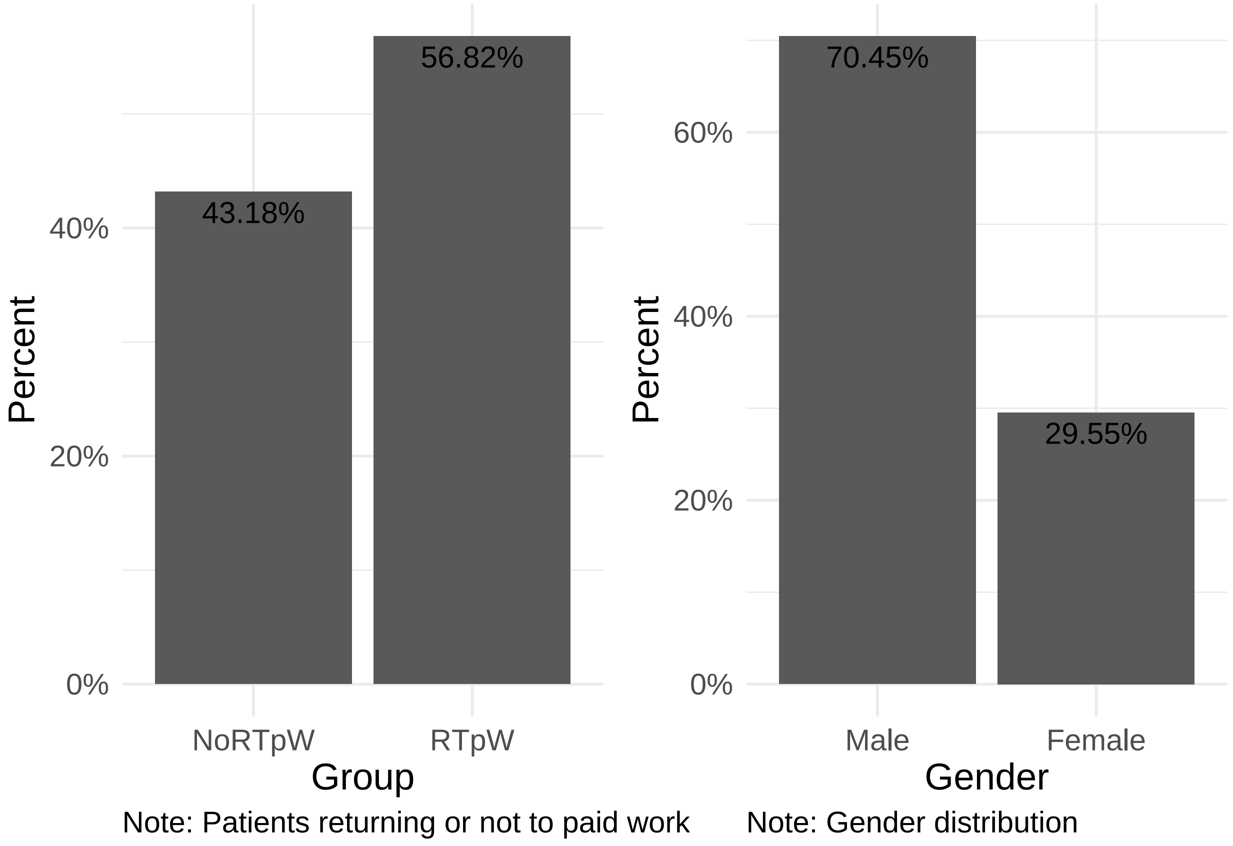

2.2 Work change from 3m to 12m follow-ups

Figure S2: Work change in percentage

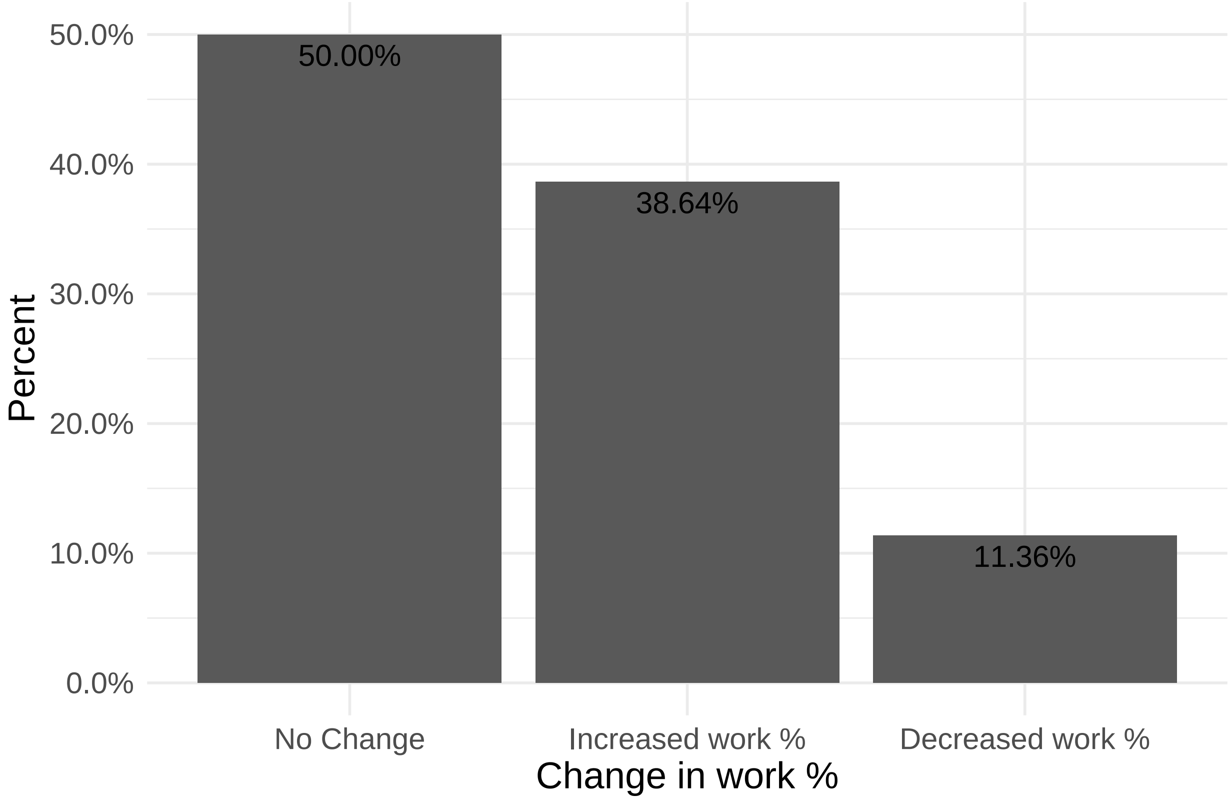

Note: Distribution of the change in work % from 3m to 12m

2.3 Discharge Destination

Table S3: Fisher's Exact Test for Count Data

	X	Df	p	Phi	OR[RTpW/NoRTpW]	2.5CI_OR	97.5CI_OR
odds ratio	0.403	1	0.525	0.074	0.4	0.034	3.005

Note:
Expected counts < 5 : 50% of cells

Figure S3: Discharge destination

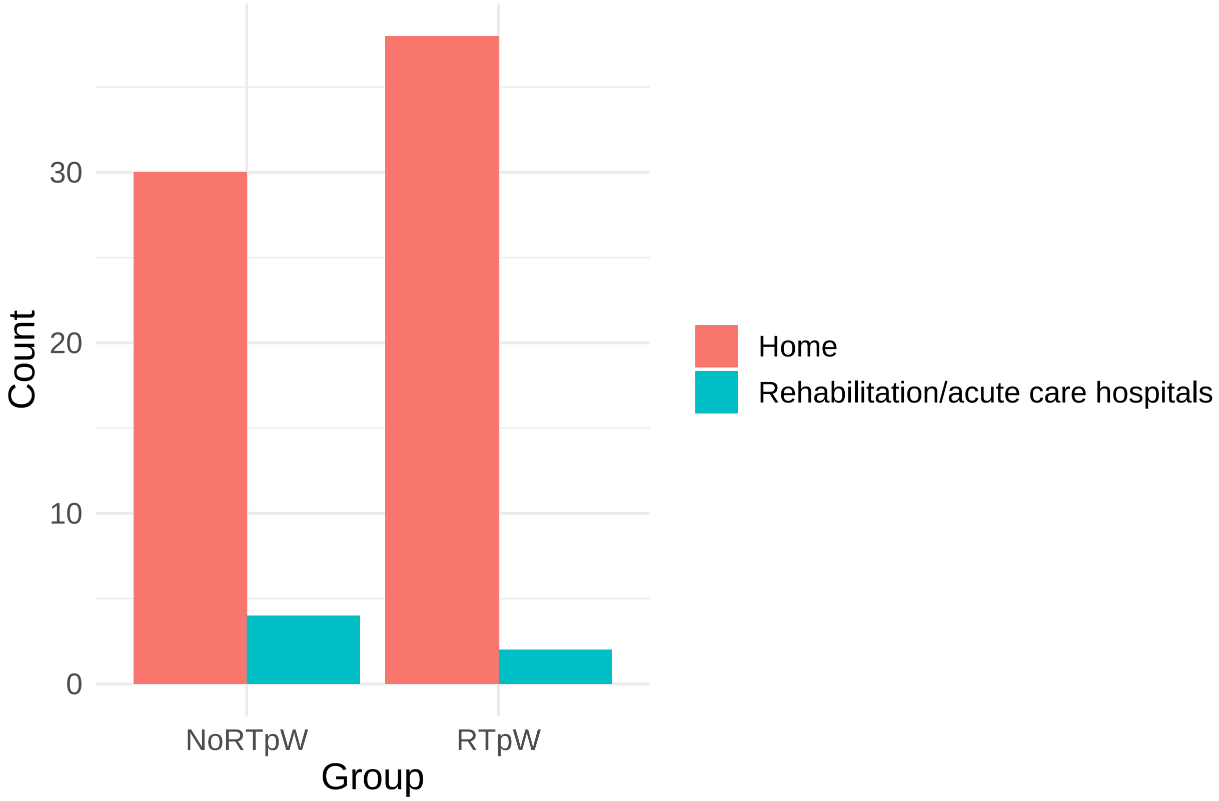

Note: Bar plot of Discharge destination distribution between groups

2.4 Etiology TOAST

Table S4: Fisher's Exact Test for Count Data

X	Df	p	Cramers_V
6.379	8	0.605	0.296

Note:
Expected counts < 5 : 66% of cells

Figure S4: Etiology TOAST

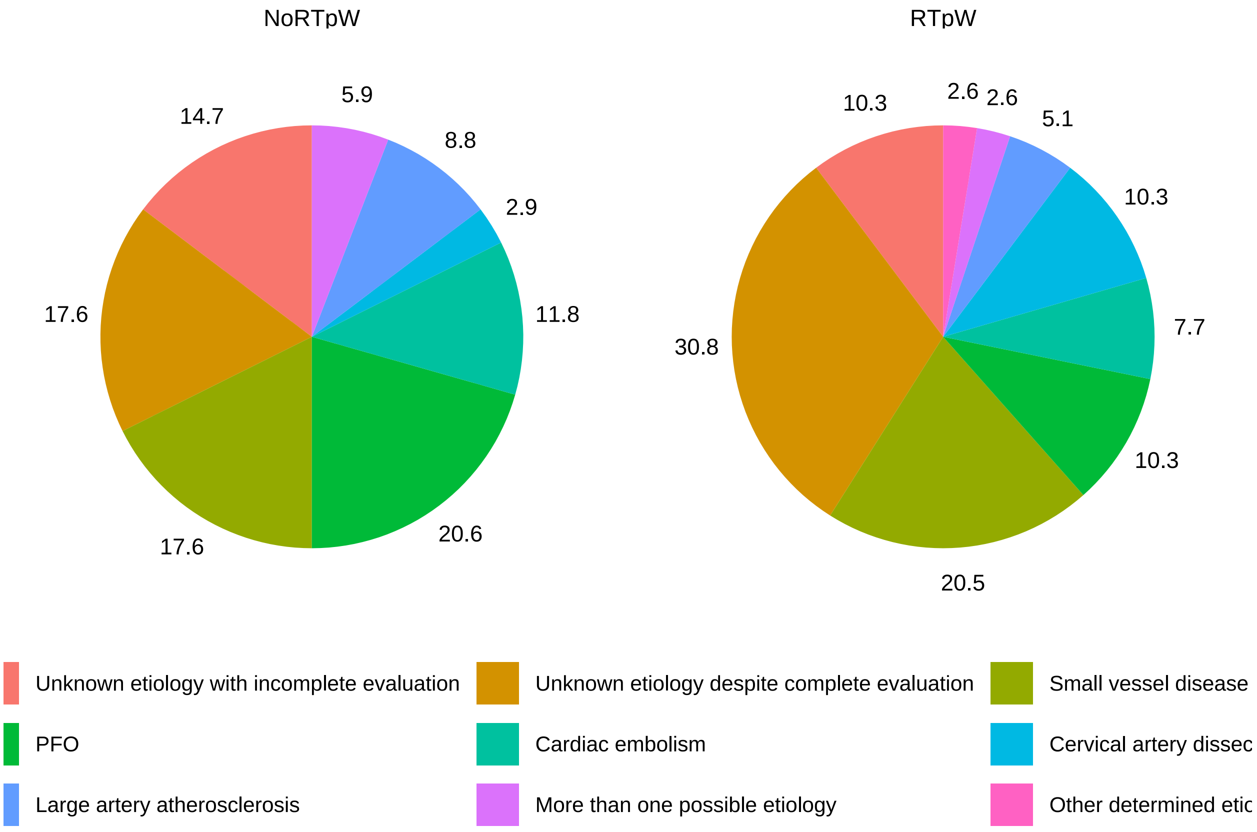

Note: Pie chart of Etiology TOAST distribution between groups

2.5 Gender

Table S5: Pearson's Chi-squared test with Yates' continuity correction

	X	Df	p	Phi	OR[RTpW/NoRTpW]	2.5CI_OR	97.5CI_OR
odds ratio	0.36	1	0.548	0.064	0.679	0.243	1.884

Note:
Expected counts < 5 : 0% of cells

Figure S5: Gender

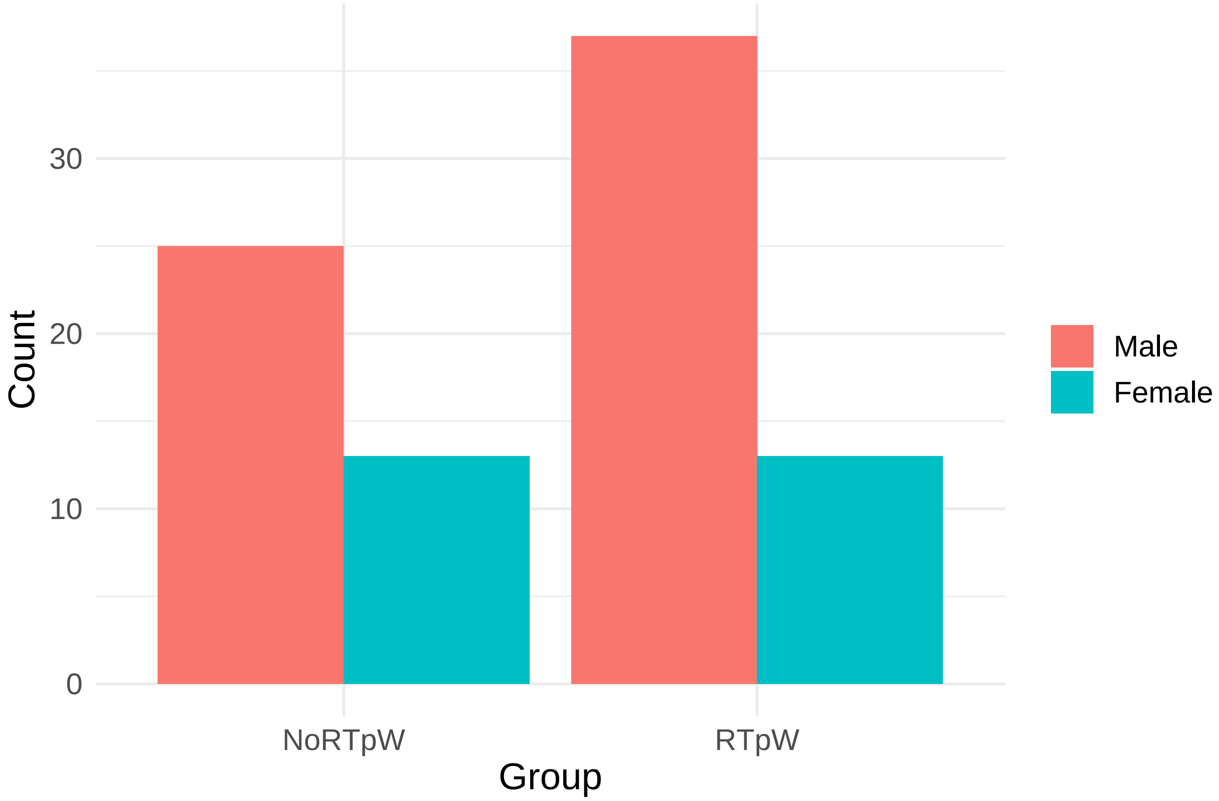

Note: Bar plot of Gender distribution between groups

2.6 Type of Work

Table S6: Pearson's Chi-squared test with Yates' continuity correction

	X	Df	p	Phi	OR[RTpW/NoRTpW]	2.5CI_OR	97.5CI_OR
odds ratio	1	1	0.317	0.107	0.585	0.222	1.523

Note:
Expected counts < 5 : 0% of cells

Figure S6: Type Of Work

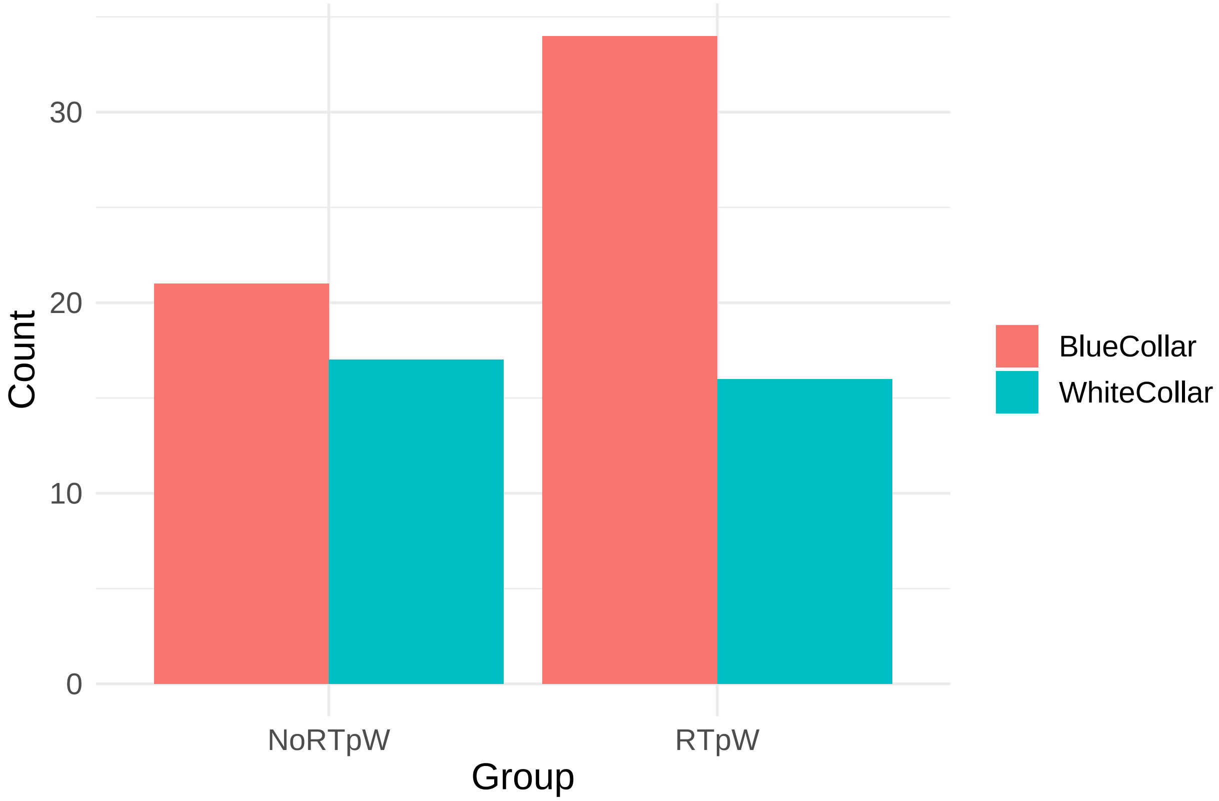

Note: Bar plot of Type Of Work distribution between groups

2.7 Event Type

Table S7: Fisher's Exact Test for Count Data

	X	Df	p	Phi	OR[RTpW/NoRTpW]	2.5CI_OR	97.5CI_OR
odds ratio	0.001	1	0.973	0.004	1.348	0.234	7.791

Note:
Expected counts < 5 : 50% of cells

Figure S7: Event Type

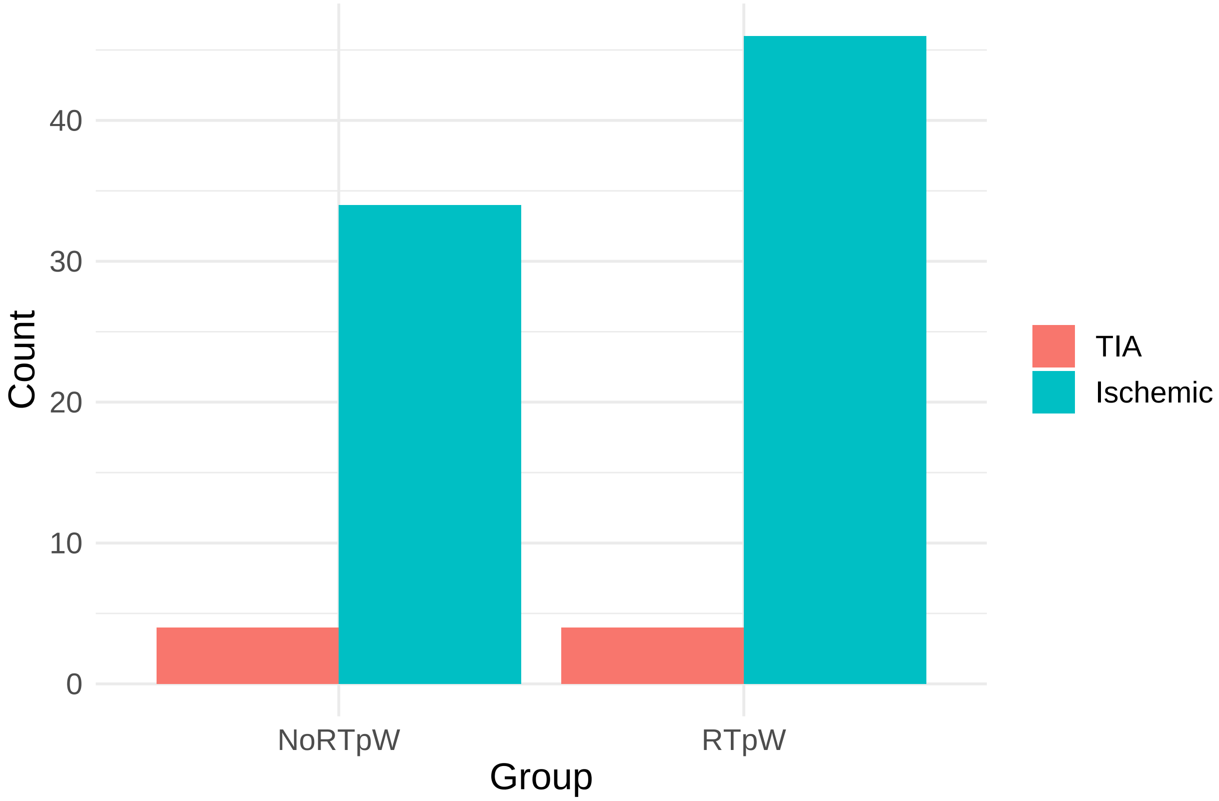

Note: Bar plot of Event Type distribution between groups

2.8 Living Situation

Table S8: Pearson's Chi-squared test with Yates' continuity correction

	X	Df	p	Phi	OR[RTpW/NoRTpW]	2.5CI_OR	97.5CI_OR
odds ratio	0.59	1	0.442	0.082	1.627	0.562	4.765

Note:
Expected counts < 5 : 0% of cells

Figure S8: Living Situation

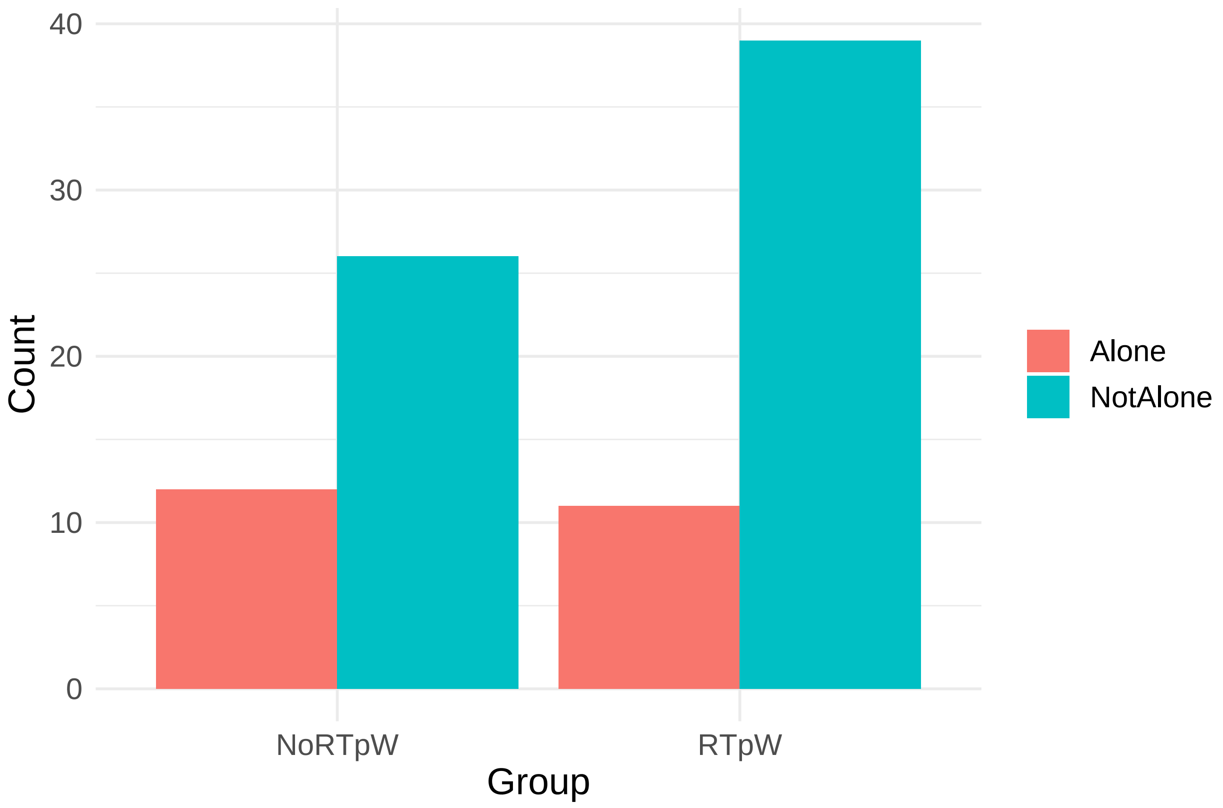

Note: Bar plot of Living Situation distribution between groups

2.9 Smoking

Table S9: Pearson's Chi-squared test with Yates' continuity correction

	X	Df	p	Phi	OR[RTpW/NoRTpW]	2.5CI_OR	97.5CI_OR
odds ratio	0.007	1	0.933	0.01	0.859	0.304	2.426

Note:
Expected counts < 5 : 0% of cells

Figure S9: Smoking

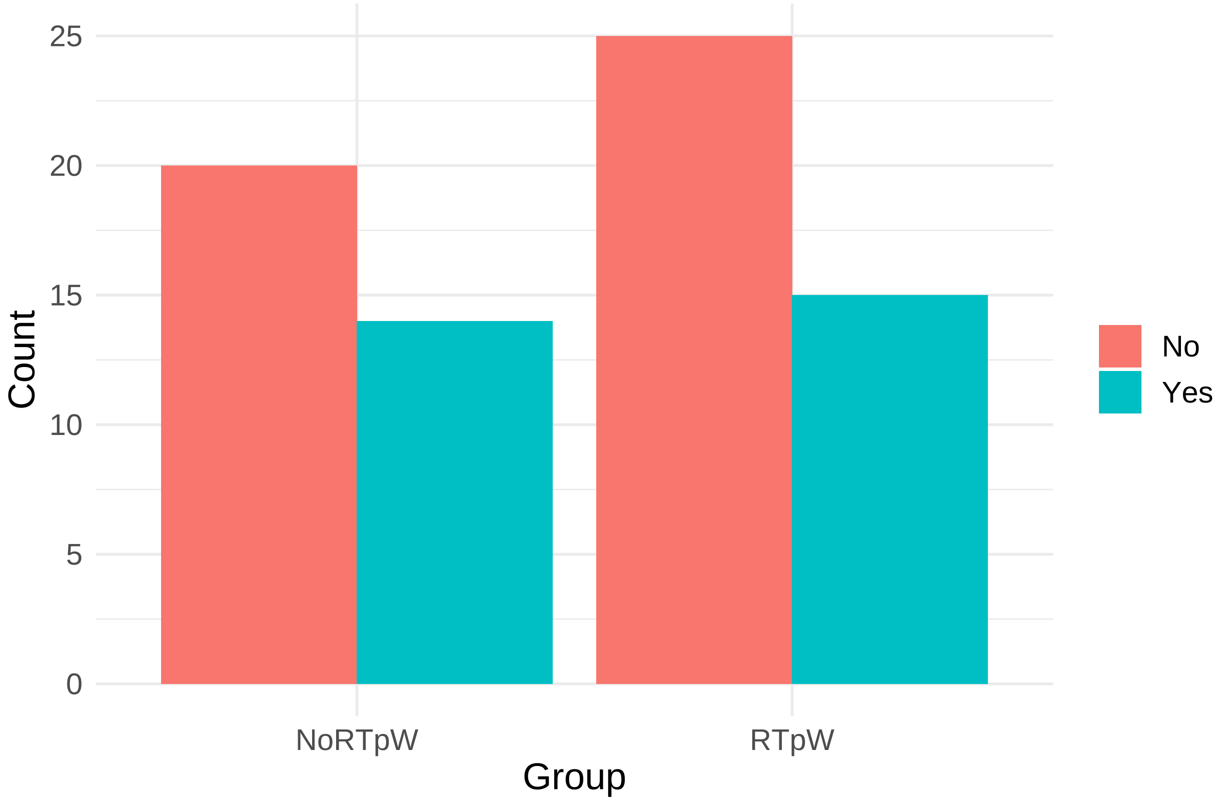

Note: Bar plot of Smoking distribution between groups

2.10 Arterial Hypertension (HTA)

Table S10: Pearson's Chi-squared test with Yates' continuity correction

	X	Df	p	Phi	OR[RTpW/NoRTpW]	2.5CI_OR	97.5CI_OR
odds ratio	0.298	1	0.585	0.062	1.422	0.528	3.894

Note:
Expected counts < 5 : 0% of cells

Figure S10: Arterial Hypertension

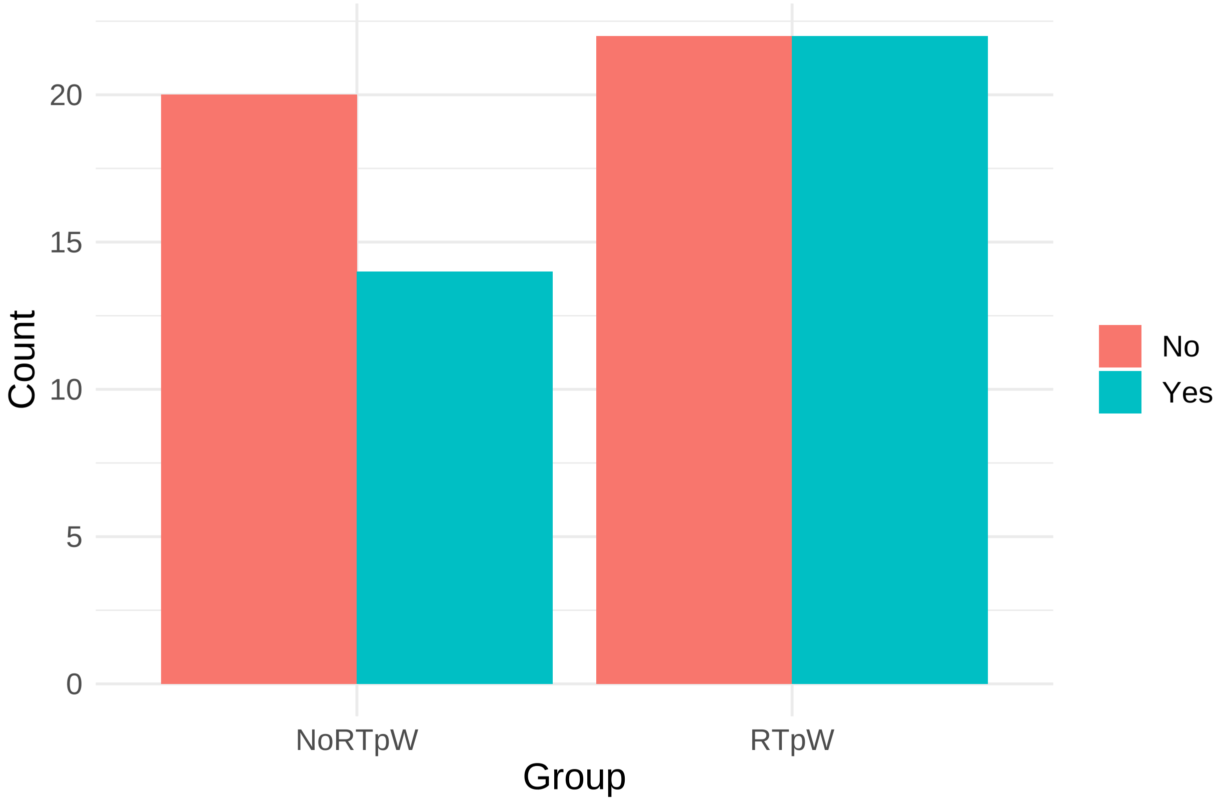

Note: Bar plot of Arterial Hypertension distribution between groups

2.11 Hyperlipidemia

Table S11: Pearson's Chi-squared test with Yates' continuity correction

	X	Df	p	Phi	OR[RTpW/NoRTpW]	2.5CI_OR	97.5CI_OR
odds ratio	6.757	1	0.009	0.287	0.218	0.056	0.711

Note:
Expected counts < 5 : 0% of cells

Figure S11: Hyperlipidemia

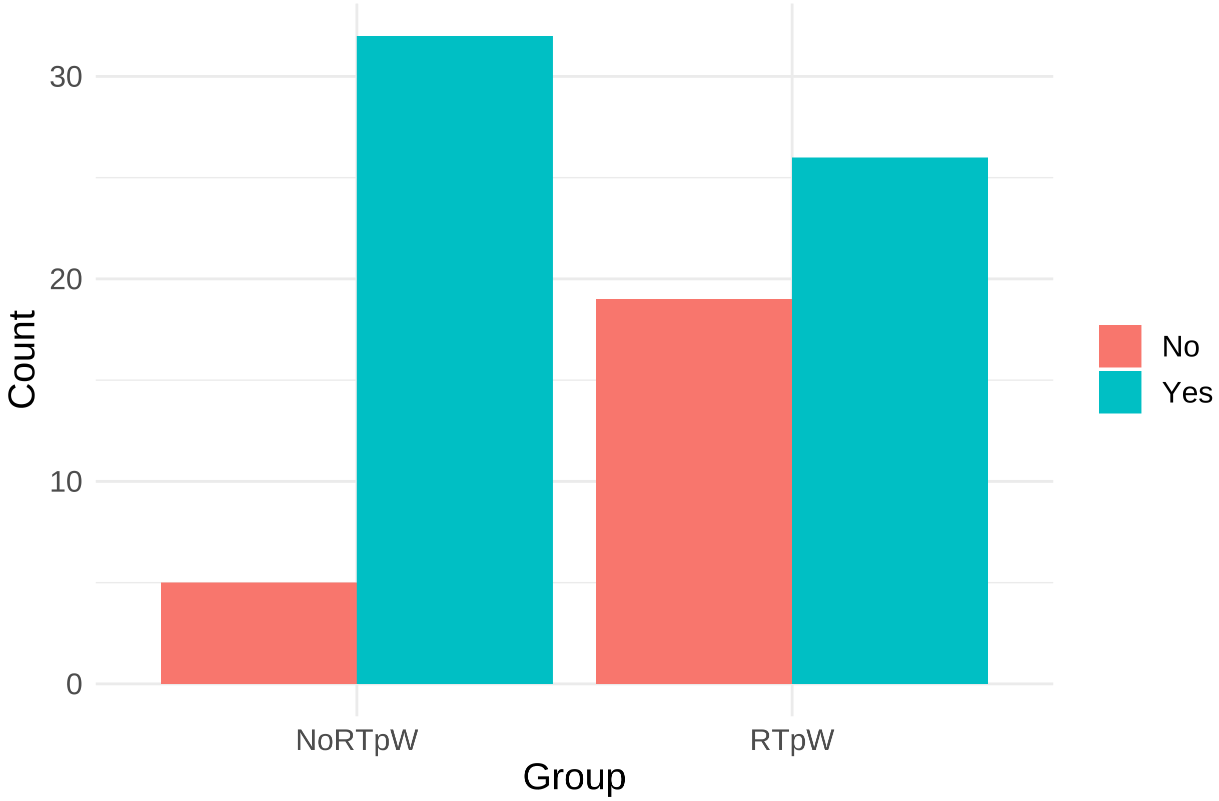

Note: Bar plot of Hyperlipidemia distribution between groups

2.12 Antilipid before Stroke

Table S12: Fisher's Exact Test for Count Data

	X	Df	p	Phi	OR[RTpW/NoRTpW]	2.5CI_OR	97.5CI_OR
odds ratio	0.015	1	0.902	0.013	0.736	0.155	3.481

Note:
Expected counts < 5 : 25% of cells

Figure S12: Antilipid before stroke

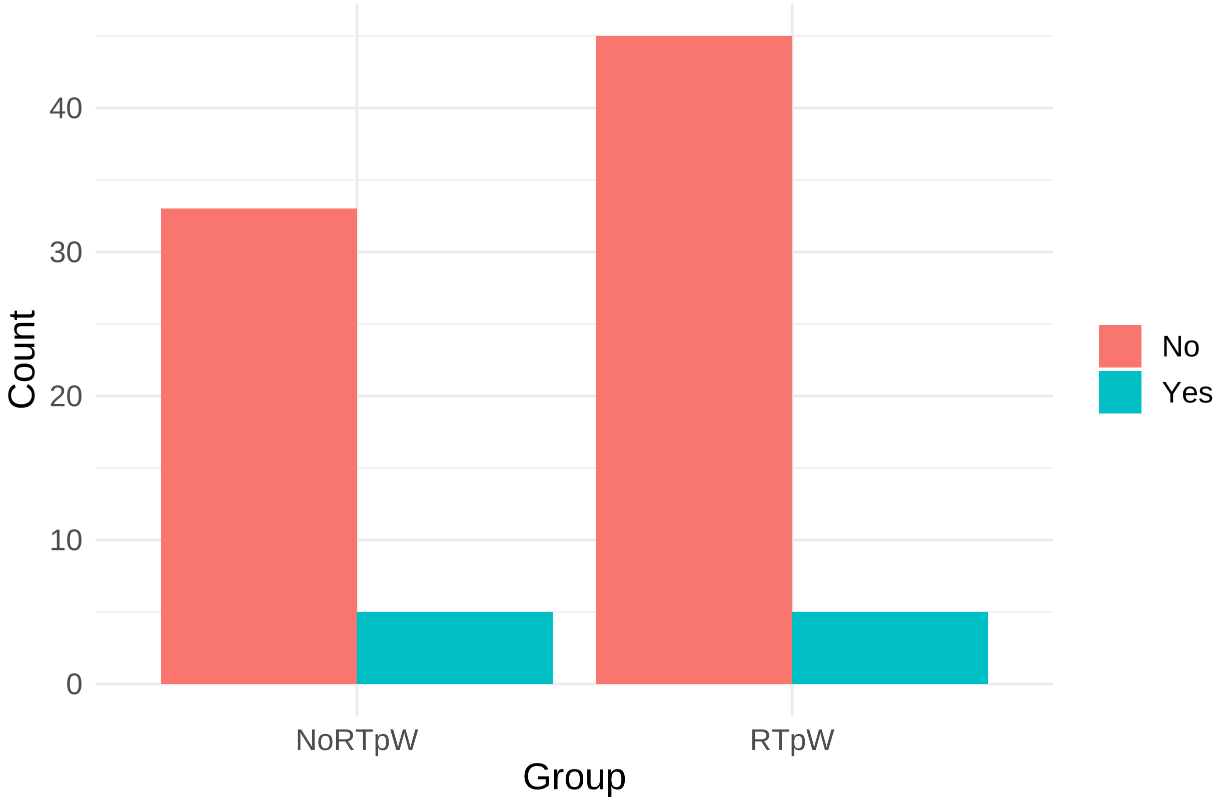

Note: Bar plot of Antilipid before stroke distribution between groups

2.13 Antilipid at Discharge

Table S13: Fisher's Exact Test for Count Data

	X	Df	p	Phi	OR[RTpW/NoRTpW]	2.5CI_OR	97.5CI_OR
odds ratio	1.52	1	0.218	0.131	0.295	0.029	1.613

Note:
Expected counts < 5 : 25% of cells

Figure S13: Antilipid at discharge

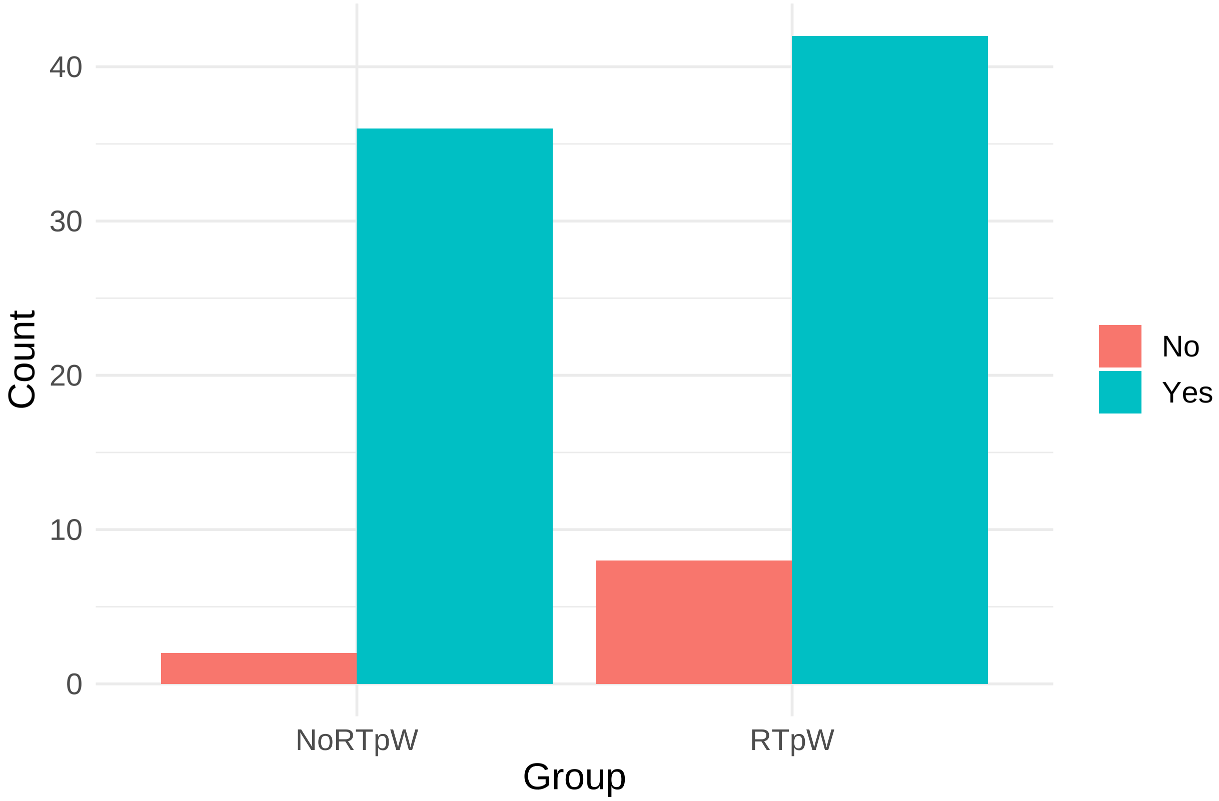

Note: Bar plot of Antilipid at discharge distribution between groups

2.14 Lesion Site

Table S14: Pearson's Chi-squared test with Yates' continuity correction

X	Df	p	Cramers_V
4.802	2	0.091	0.236

Note:
Expected counts < 5 : 0% of cells

Figure S14: Lesion Site

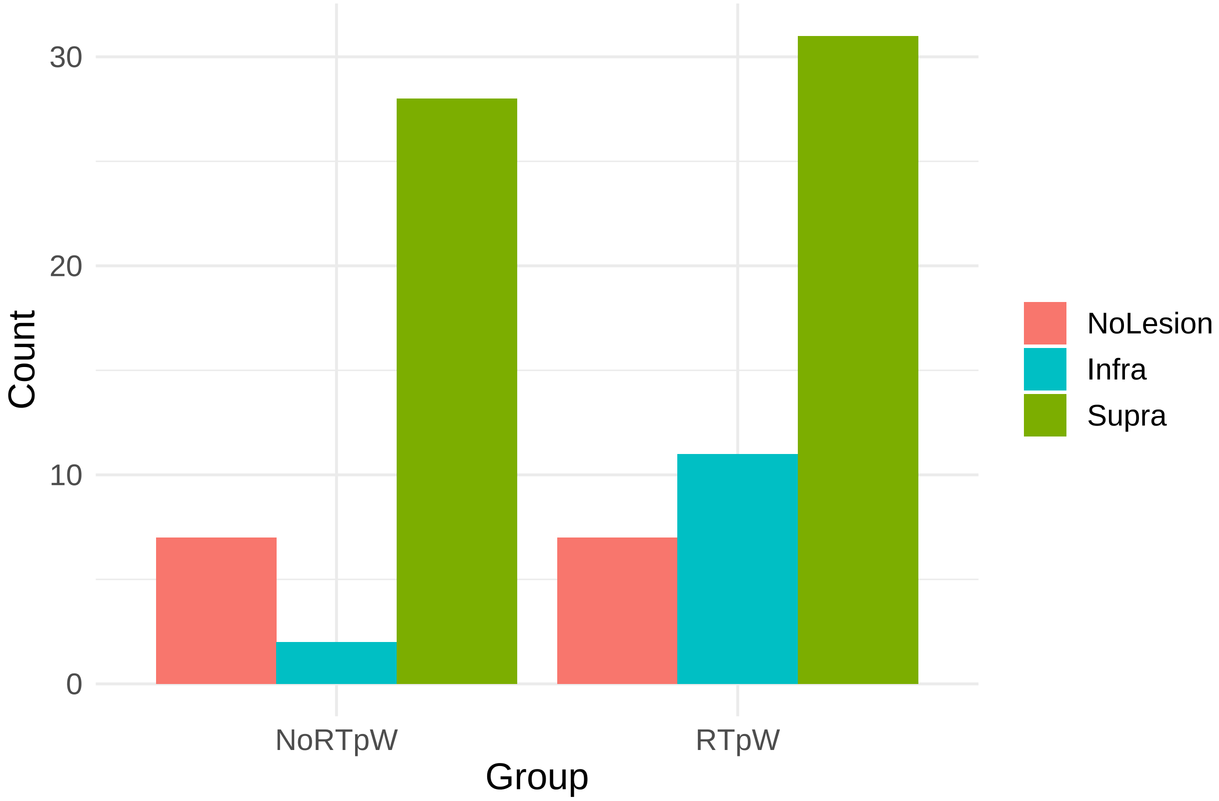

Note: Bar plot of Lesion Site distribution between groups

2.15 Thyroid-Stimulating Hormone (TSH)

Table S15: Wilcoxon rank sum test with continuity correction

Stat	p	z	r	2.5CI	97.5CI
211	0.927	-0.092	0.011	-0.64	0.82

Figure S15: Thyroid-Stimulating Hormone

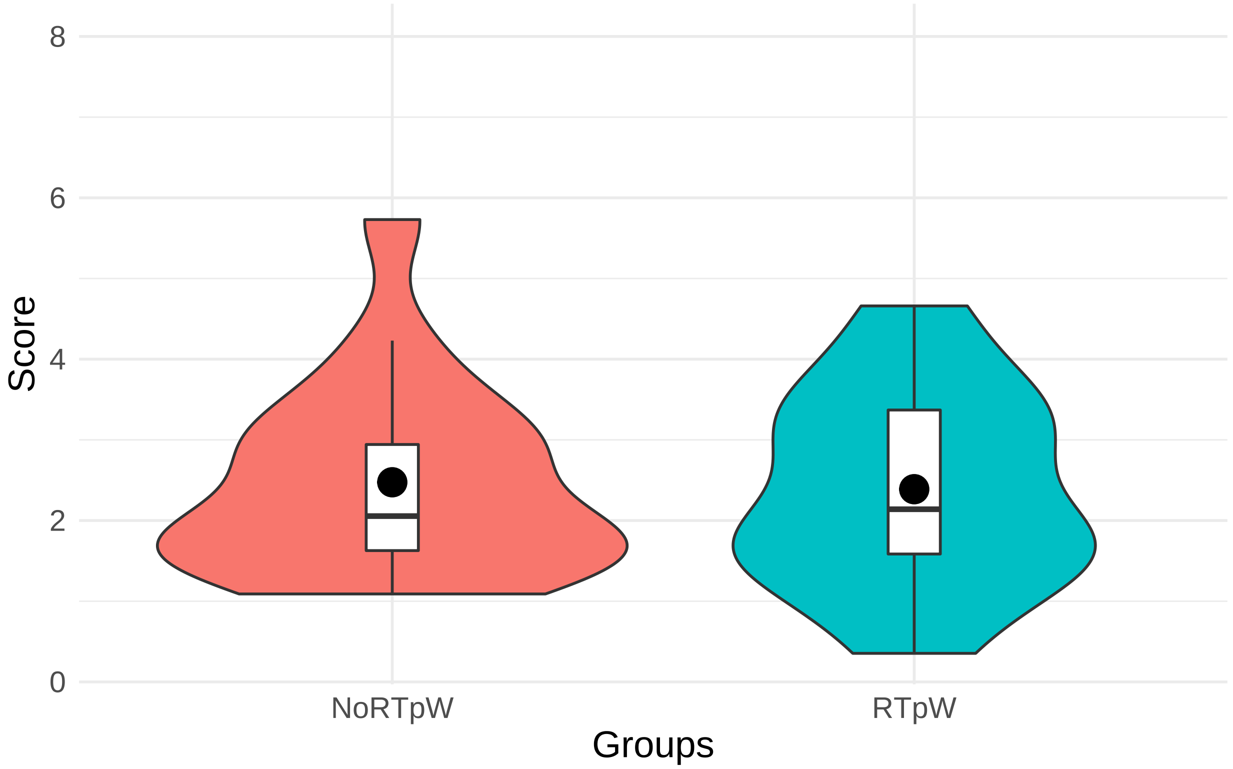

Note: Violin plot of Thyroid-Stimulating Hormone (TSH) distribution between groups
[*]p<.05; [**]p<.01; [***]p<.001

2.16 Duration of Hospitalization

Table S16: Wilcoxon rank sum test with continuity correction

Stat	p	z	r	2.5CI	97.5CI
836	0.092	-1.687	0.18	-0.175	1.749

Figure S16: Duration hospitalization

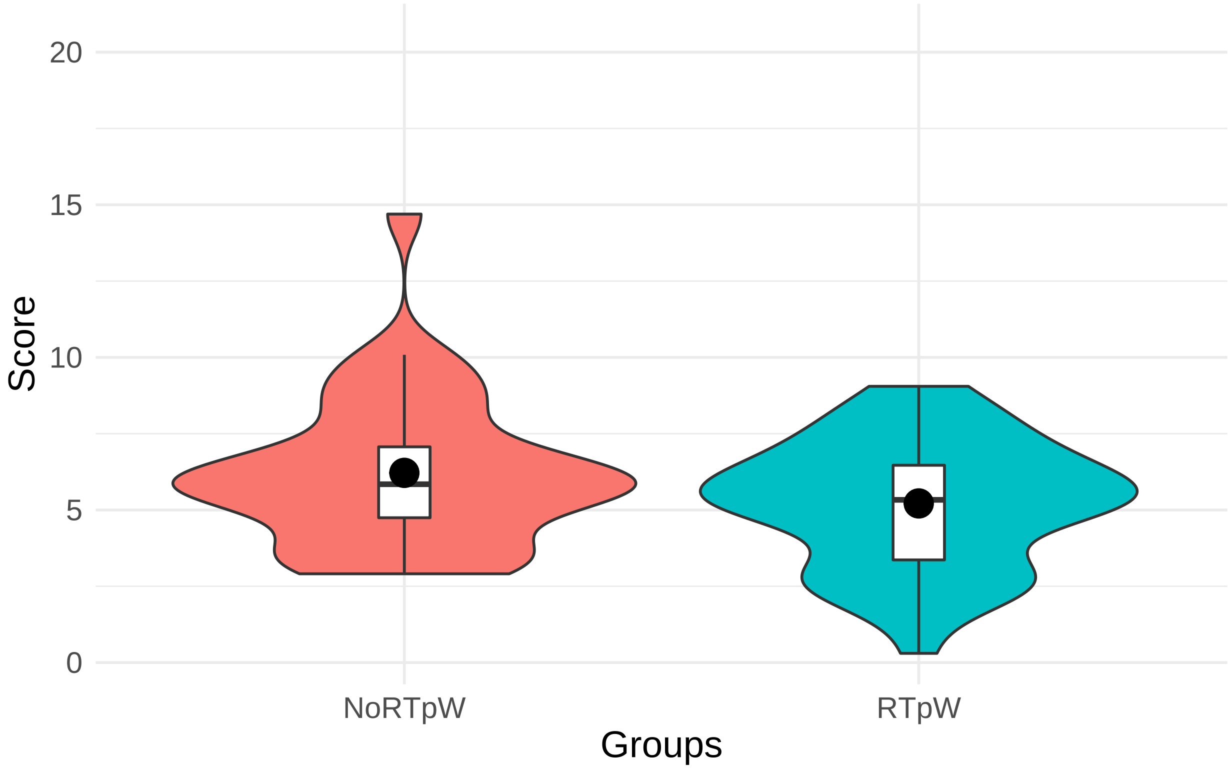

Note: Violin plot of Duration hospitalization distribution between groups
[*]p<.05; [**]p<.01; [***]p<.001

2.17 Age

Table S17: Wilcoxon rank sum test with continuity correction

Stat	p	z	r	2.5CI	97.5CI
1006	0.64	-0.468	0.05	-3	4

Figure S17: Age

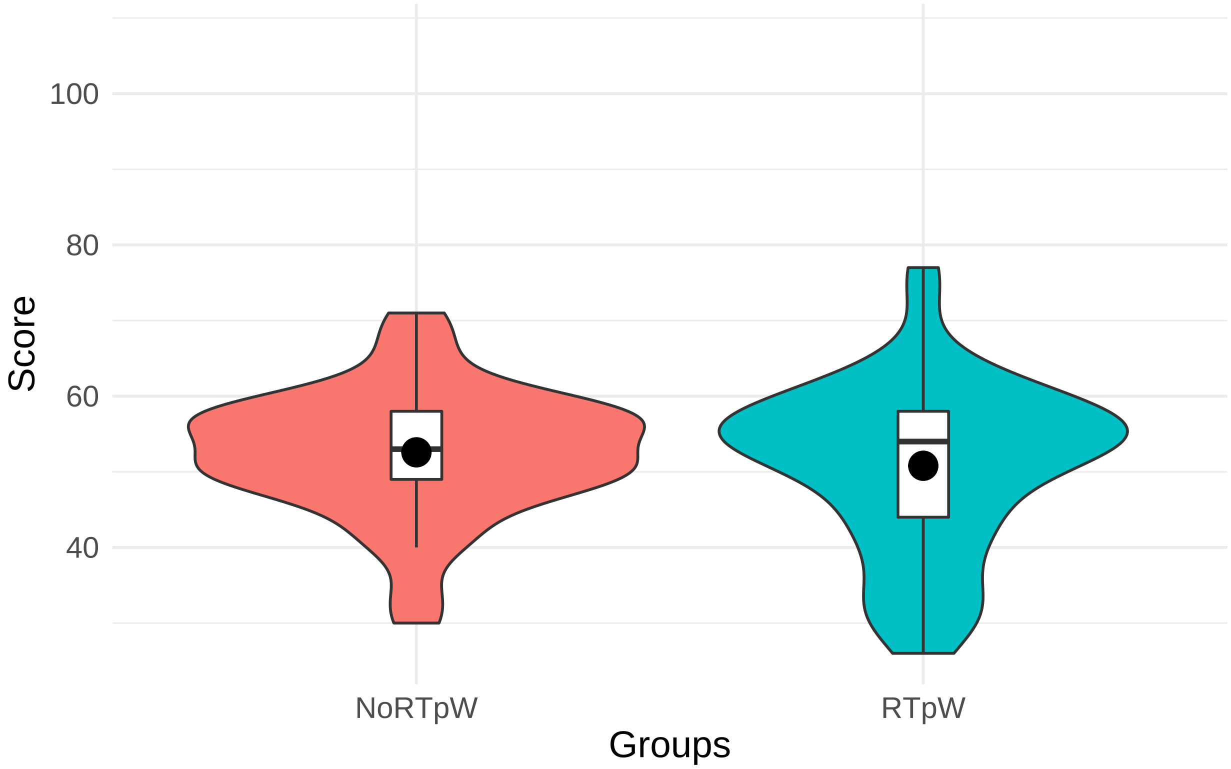

Note: Violin plot of Age distribution between groups
[*]p<.05; [**]p<.01; [***]p<.001

2.18 BMI

Table S18: Wilcoxon rank sum test with continuity correction

Stat	p	z	r	2.5CI	97.5CI
1058	0.365	-0.906	0.097	-1.1	3.3

Figure S18: BMI

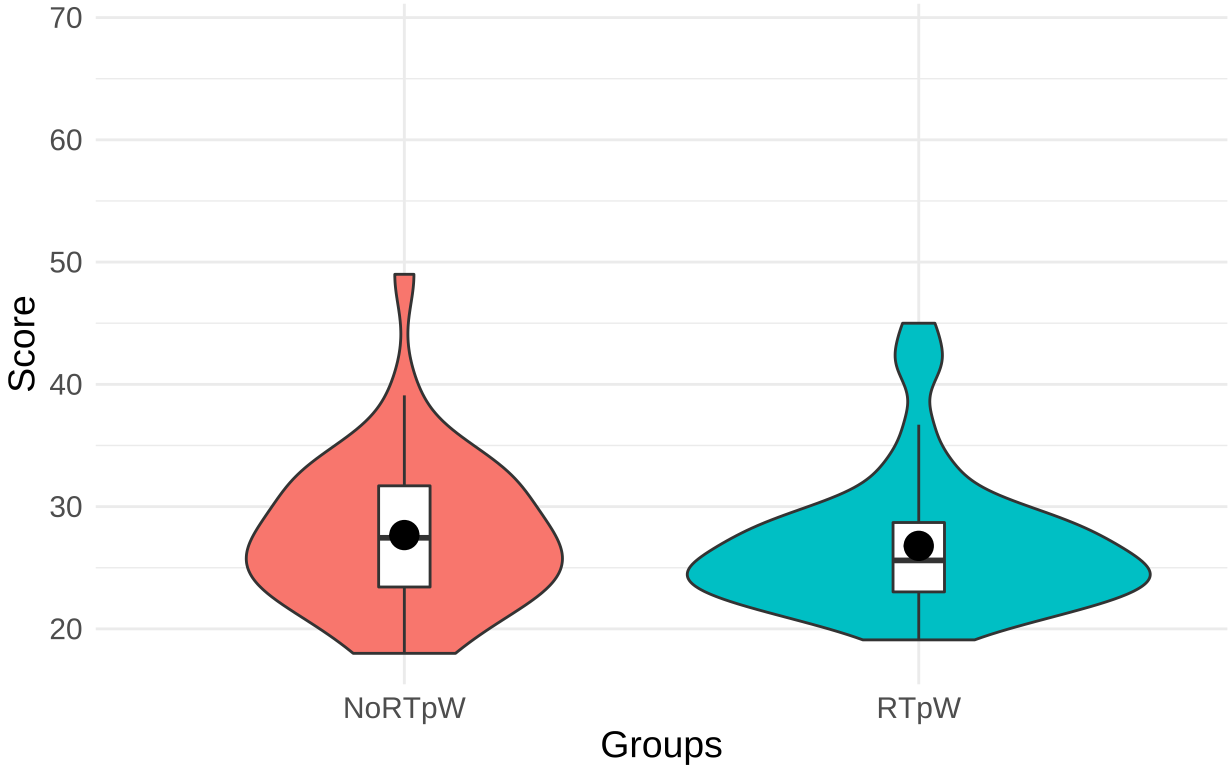

Note: Violin plot of BMI distribution between groups
[*]p<.05; [**]p<.01; [***]p<.001

2.19 LDL-Cholesterol (LDL-C)

Table S19: Welch Two Sample t-test

t	Df	p	HedgesG	2.5CI	97.5CI
0.305	82	0.761	0.066	-0.368	0.502

Figure S19: LDL-Cholesterol

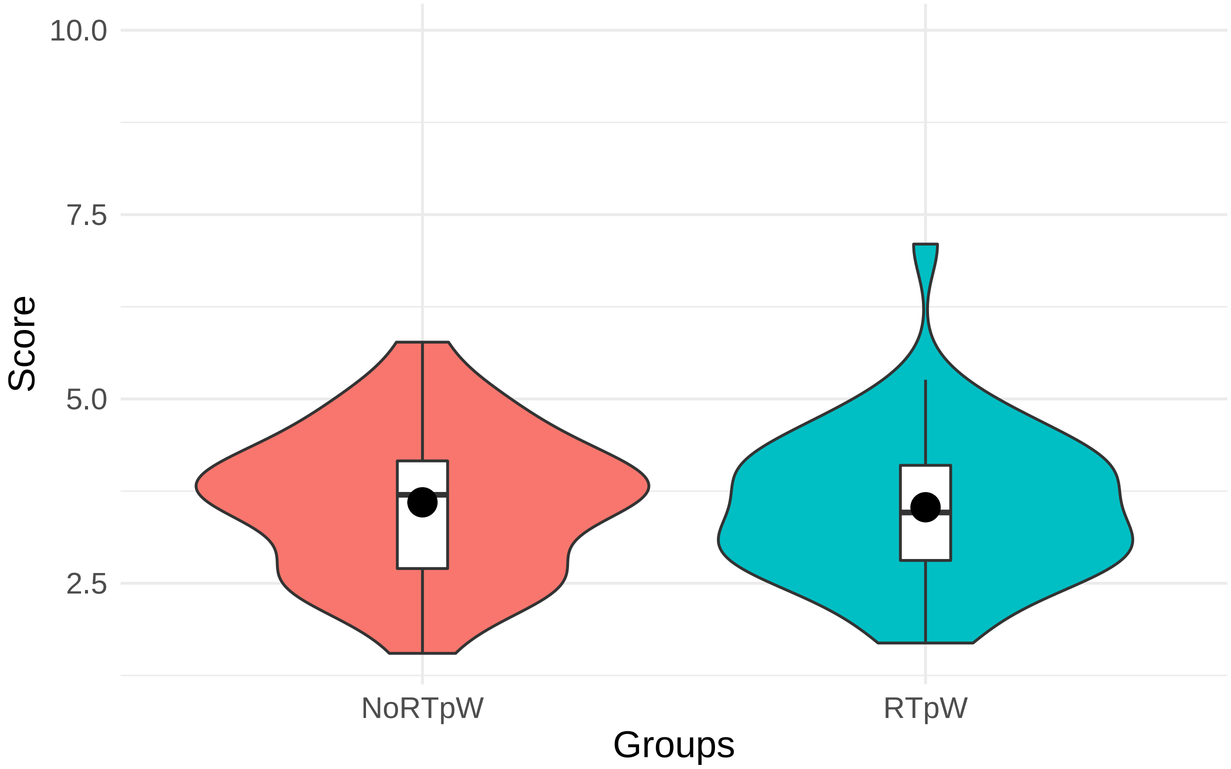

Note: Violin plot of LDL-Cholesterol (LDL-C) distribution between groups
[*]p<.05; [**]p<.01; [***]p<.001

2.20 Total Cholesterol

Table S20: Welch Two Sample t-test

t	Df	p	HedgesG	2.5CI	97.5CI
-0.063	81	0.95	-0.014	-0.475	0.446

Figure S20: Total Cholesterol

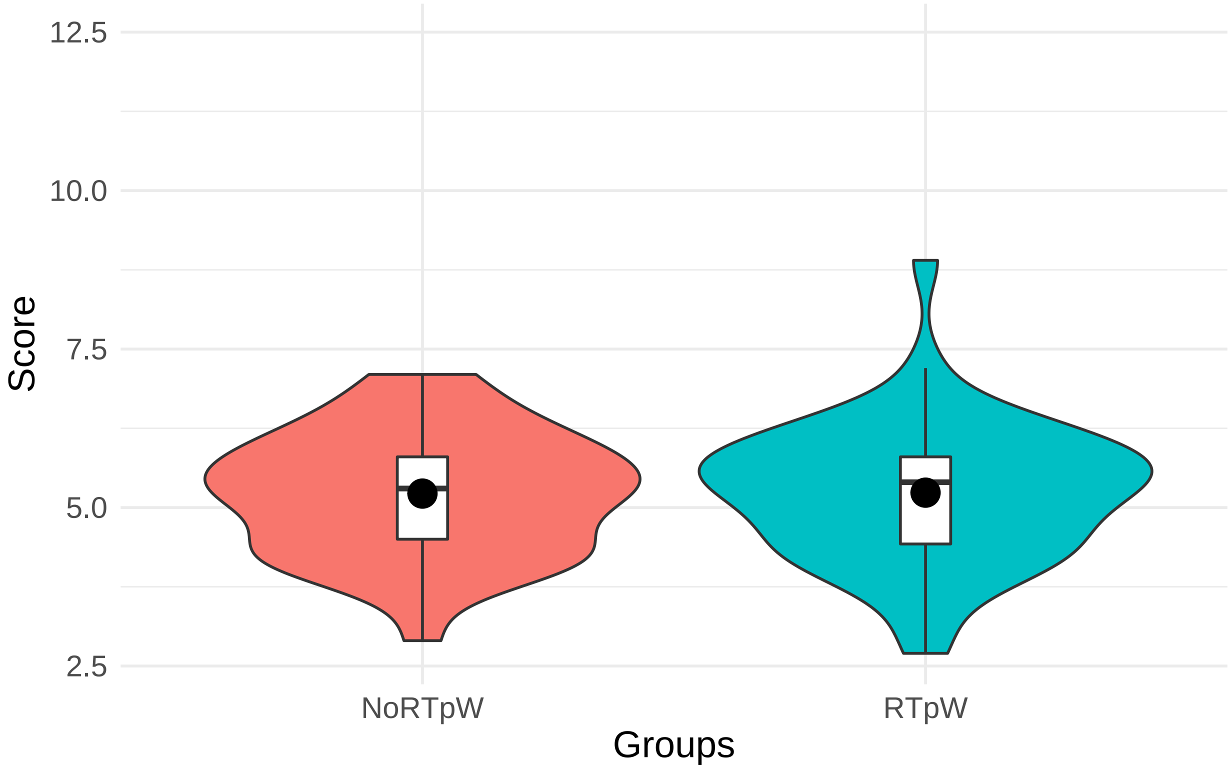

Note: Violin plot of Total Cholesterol distribution between groups
[*]p<.05; [**]p<.01; [***]p<.001

3 ROBUST ANOVAs

3.1 NIHSS

Table S21: ROBUST ANOVA using trimmed means

	F	Df1	Df2	p
Between	4.738	1	19.998	0.042
Within	11.485	1	21.420	0.003
Interaction	2.139	1	21.420	0.158

Figure S21: NIHSS

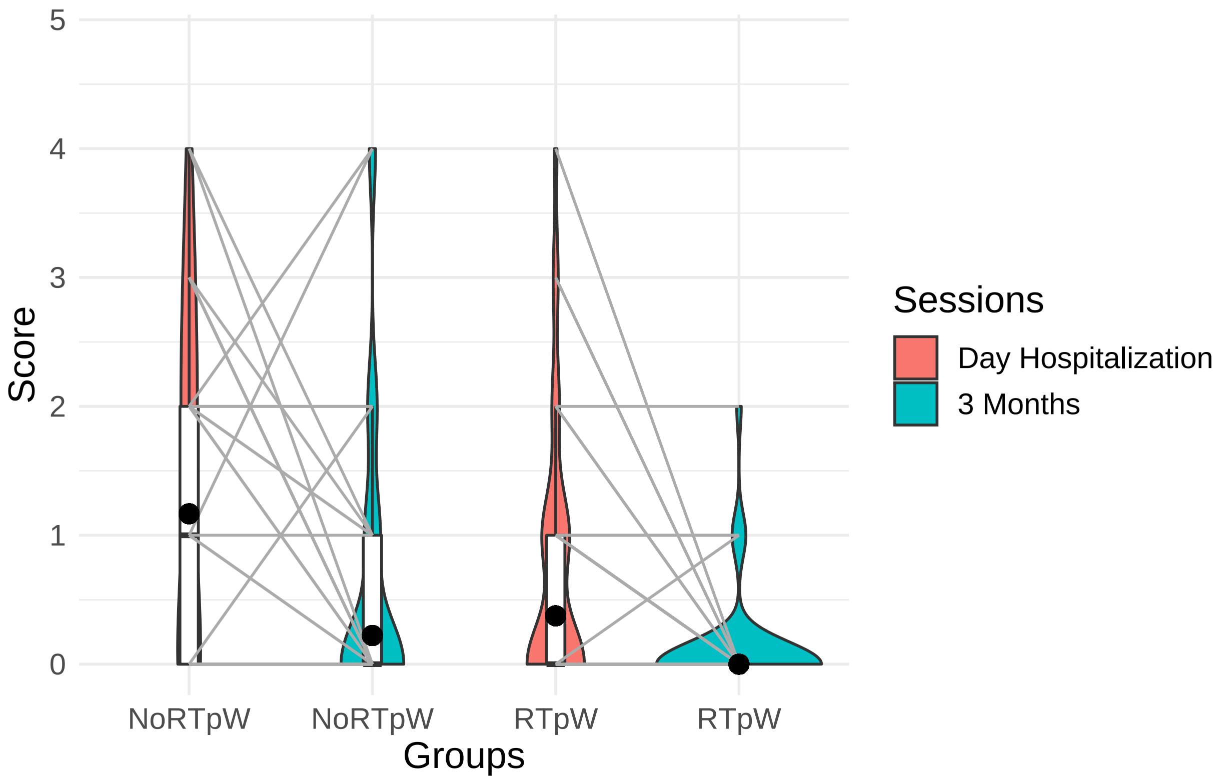

Note: Violin plot of NIHSS distribution between groups
[*]p<.05; [**]p<.01; [***]p<.001

3.2 Montreal Cognitive Assessment (MoCA)

Table S22: ROBUST ANOVA using trimmed means

	F	Df1	Df2	p
Between	0.819	1	39.41	0.371
Within	2.029	1	39.97	0.162
Interaction	0.249	1	39.97	0.621

Figure S22: Montreal Cognitive Assessment (MoCA)

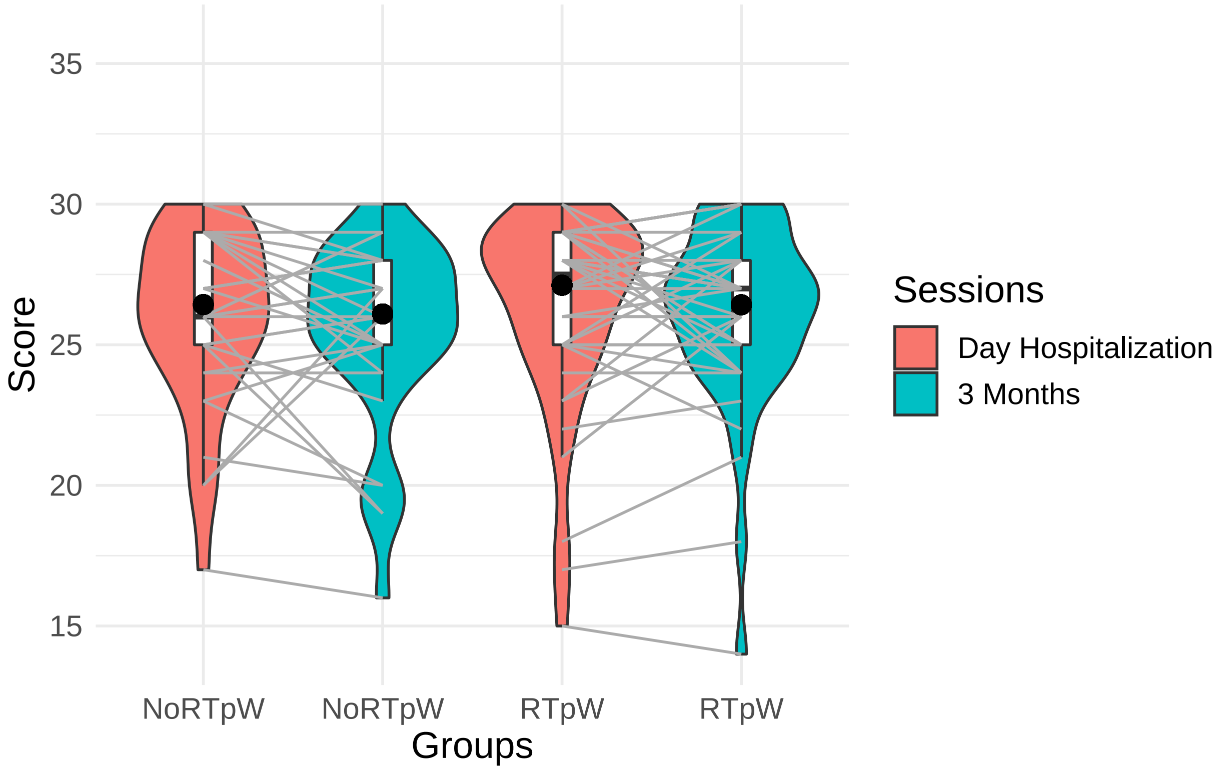

Note: Violin plot of Montreal Cognitive Assessment (MoCA) distribution between groups
[*]p<.05; [**]p<.01; [***]p<.001

3.3 Hospital Anxiety and Depression scale (HAD)

Table S23: ROBUST ANOVA using trimmed means

	F	Df1	Df2	p
Between	8.380	1	38.265	0.006
Within	2.308	1	33.097	0.138
Interaction	9.077	1	33.097	0.005

Table S24: Yuen's test using trimmed means

	t	Df	p	d	2.5CI	97.5CI
RTpW:onset Vs NoRTpW:onset (B)	0.846	36.754	0.403	0.140	-1.794	4.365
RTpW:3m Vs NoRTpW:3m (B)	3.742	37.109	0.001	0.567	3.145	10.569
RTpW:onset Vs RTpW:3m (W)	9.154	41.000	0.000	0.878	5.474	8.573
NoRTpW:onset Vs NoRTpW:3m(W)	10.738	39.000	0.000	0.925	8.867	12.983

Figure S23: Hospital Anxiety and Depression scale (HAD)

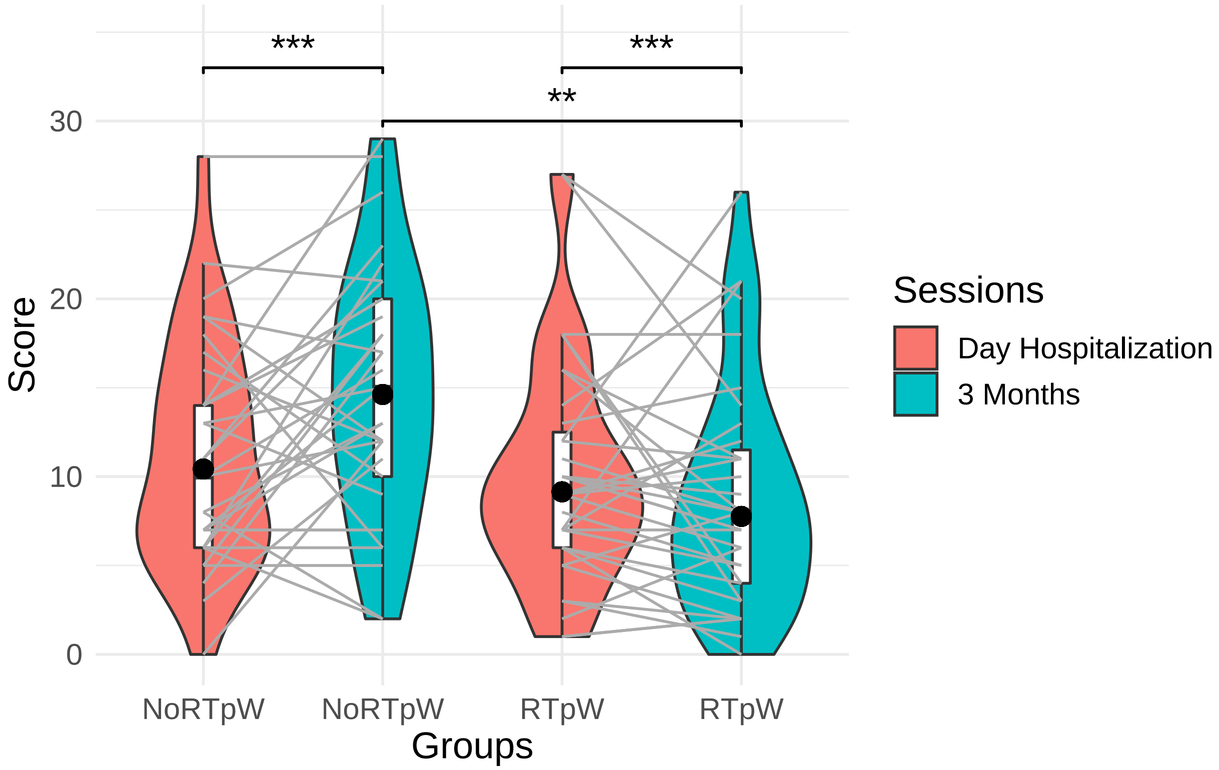

Note: Violin plot of Hospital Anxiety and Depression scale (HAD) distribution between g
[*]p<.05; [**]p<.01; [***]p<.001

3.4 Fatigue Impact Scale (FIS) Cognitive

Table S25: ROBUST ANOVA using trimmed means

	F	Df1	Df2	p
Between	16.720	1	31.062	0.000
Within	17.423	1	31.471	0.000
Interaction	13.875	1	31.471	0.001

Table S26: Yuen's test using trimmed means

	t	Df	p	d	2.5CI	97.5CI
RTpW:onset Vs NoRTpW:onset (B)	1.240	28.603	0.225	0.242	-6.203	25.282
RTpW:3m Vs NoRTpW:3m (B)	5.471	33.839	0.000	0.699	26.565	57.977
RTpW:onset Vs RTpW:3m (W)	6.465	37.000	0.000	0.750	14.599	27.927
NoRTpW:onset Vs NoRTpW:3m(W)	8.248	33.000	0.000	0.848	35.423	58.618

Figure S24: Fatigue Impact Scale (FIS) Cognitive

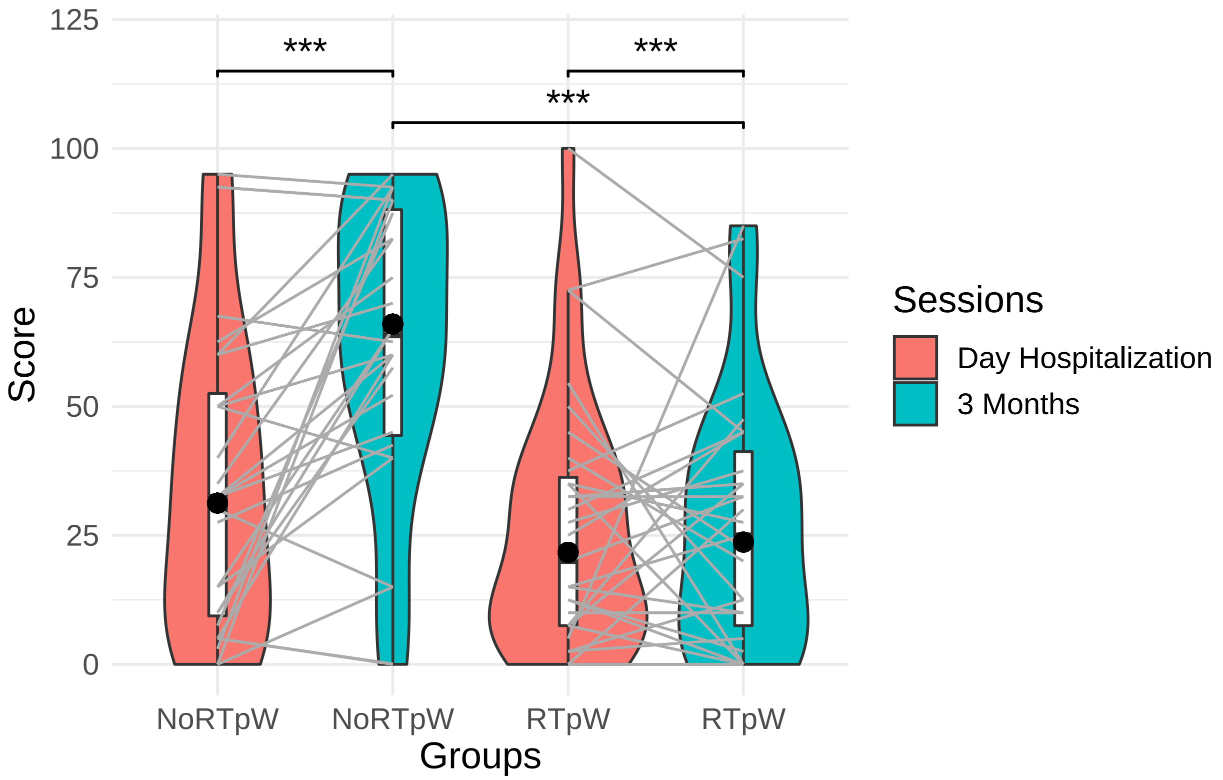

Note: Violin plot of Fatigue Impact Scale (FIS) Cognitive distribution between groups
[*]p<.05; [**]p<.01; [***]p<.001

3.5 FIS Physical

Table S27: ROBUST ANOVA using trimmed means

	F	Df1	Df2	p
Between	9.650	1	33.115	0.004
Within	8.516	1	33.716	0.006
Interaction	5.281	1	33.716	0.028

Table S28: Yuen's test using trimmed means

	t	Df	p	d	2.5CI	97.5CI
RTpW:onset Vs NoRTpW:onset (B)	0.873	31.253	0.389	0.157	-8.633	21.557
RTpW:3m Vs NoRTpW:3m (B)	3.948	25.728	0.001	0.617	13.373	42.450
RTpW:onset Vs RTpW:3m (W)	7.588	37.000	0.000	0.846	20.938	36.194
NoRTpW:onset Vs NoRTpW:3m(W)	11.948	33.000	0.000	0.913	39.709	56.008

Figure S25: FIS Physical

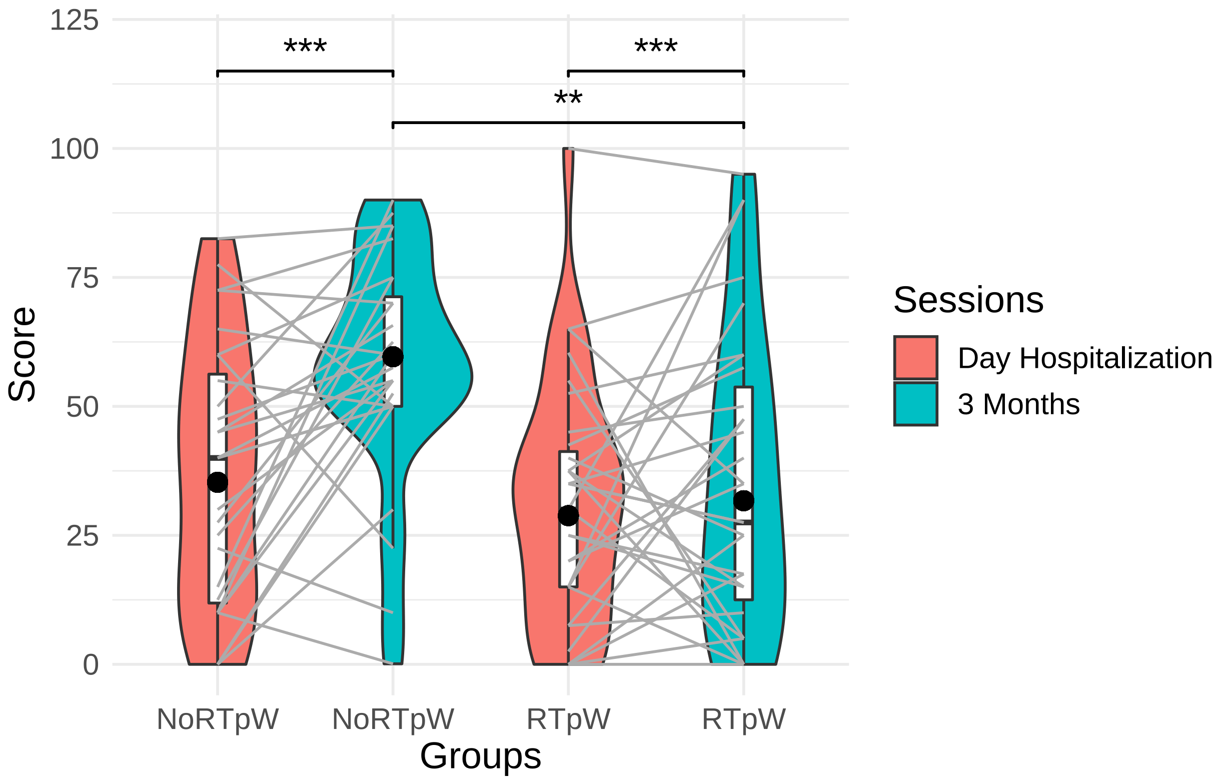

Note: Violin plot of FIS Physical distribution between groups
[*]p<.05; [**]p<.01; [***]p<.001

3.6 FIS Psychosocial

Table S29: ROBUST ANOVA using trimmed means

	F	Df1	Df2	p
Between	21.809	1	33.846	0.000
Within	13.847	1	33.980	0.001
Interaction	22.714	1	33.980	0.000

Table S30: Yuen's test using trimmed means

	t	Df	p	d	2.5CI	97.5CI
RTpW:onset Vs NoRTpW:onset (B)	1.226	33.397	0.229	0.242	-4.308	17.400
RTpW:3m Vs NoRTpW:3m (B)	6.199	33.768	0.000	0.752	24.973	49.343
RTpW:onset Vs RTpW:3m (W)	7.969	37.000	0.000	0.869	16.544	27.825
NoRTpW:onset Vs NoRTpW:3m(W)	10.946	33.000	0.000	0.897	36.118	52.610

Figure S26: FIS Psychosocial

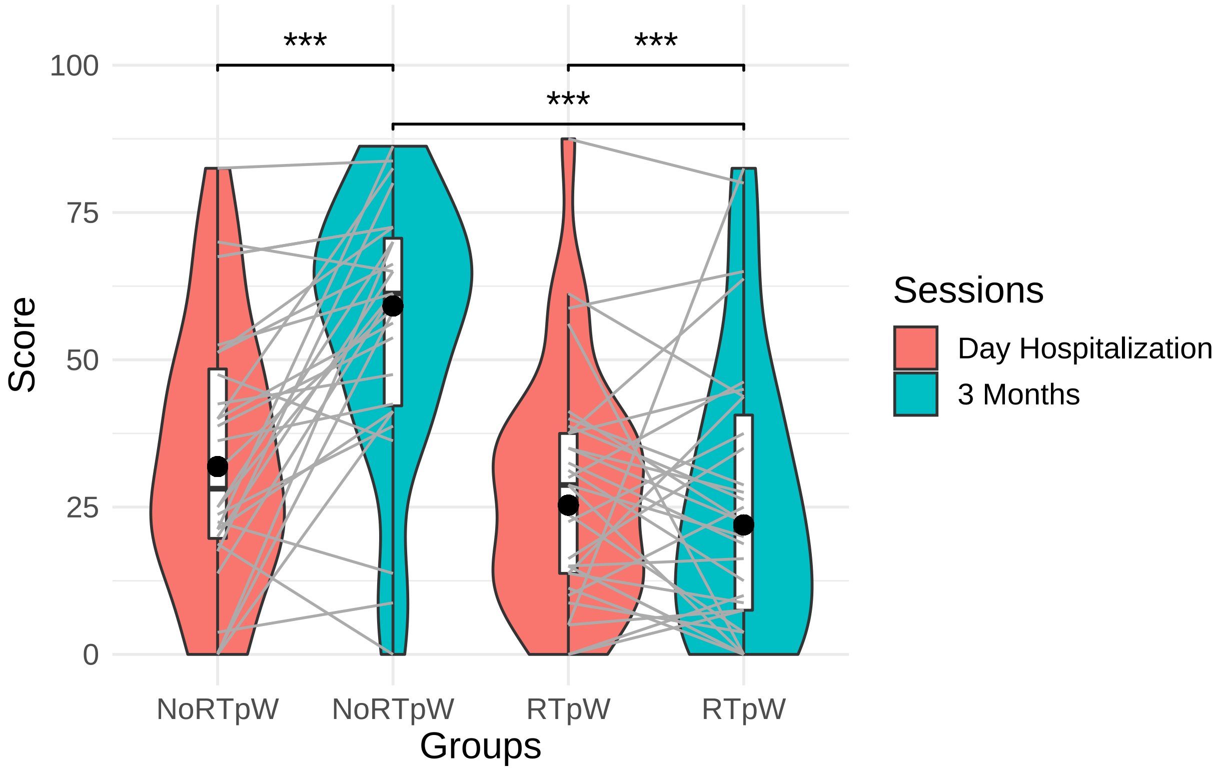

Note: Violin plot of FIS Psychosocial distribution between groups
[*]p<.05; [**]p<.01; [***]p<.001

4 DESCRIPTIVES AND STATISTICAL TESTS SUMMARY (extended Table 1 from manuscript)

Table S31: Extended table 1 from manuscript

	Overall			NoRTpW			RTpW			Statistics						
	TMean/Median	SD/IQR	N	TMean/Median	SD/IQR	N	TMean/Median	SD/IQR	N	Stat	Df	z	*p*	ES/OR	2.25CI	97.5CI
Demographics																
Gender:Male			62			25			37							
Gender:Female			26			13			13	0.36	1		0.55	0.68	0.24	1.88
Type of Work:Blue collar			55			21			34							
Type of Work:White collar			33			17			16	1.00	1		0.32	0.58	0.22	1.52
Living situation:Alone			23			12			11							
Living situation:Not alone			65			26			39	0.59	1		0.44	1.63	0.56	4.76
Age	54.00	10.50	88	53.00	9.00	38	54.00	14.00	50	1006.00		-0.47	0.64	0.05	-3.00	4.00
Cardiovascular risk factors and comorbidities																
Hypertension:No			42			20			22							
Hypertension:Yes			36			14			22	0.30	1		0.58	1.42	0.53	3.89
**Hyperlipidemia:No**			**24**			**5**			**19**							
**Hyperlipidemia:Yes**			**58**			**32**			**26**	**6.76**	**1**		**0.01**	**0.22**	**0.06**	**0.71**
Smoking:No			45			20			25							
Smoking:Yes			29			14			15	0.01	1		0.93	0.86	0.30	2.43
LDL Cholesterol	3.56	0.99	84	3.60	0.97	37	3.53	1.02	47	0.31	82		0.76	0.07	-0.37	0.50
Total Cholesterol	5.23	1.04	83	5.22	0.98	37	5.23	1.10	46	-0.06	81		0.95	-0.01	-0.48	0.45
BMI	25.85	6.32	88	27.45	8.28	38	25.60	5.68	50	1058.00		-0.91	0.37	0.10	-1.10	3.30
TSH	2.07	1.67	41	2.05	1.31	18	2.14	1.79	23	211.00		-0.09	0.93	0.01	-0.64	0.82
Medications prior to stroke onset																
Antilipid pre:No			78			33			45							
Antilipid pre:Yes			10			5			5	0.02	1		0.90	0.74	0.16	3.48
Antilipid at discharge:No			10			2			8							
Antilipid at discharge:Yes			78			36			42	1.52	1		0.22	0.30	0.03	1.61
Cerebrovascular event																
Event type:TIA			8			4			4							
Event type:MIS			80			34			46	0.00	1		0.97	1.35	0.23	7.79
Discharge destination:[1]			68			30			38							
Discharge destination:[2]			6			4			2	0.40	1		0.53	0.40	0.03	3.01
Etiology TOAST:[a]			9			5			4							
Etiology TOAST:[b]			18			6			12							
Etiology TOAST:[c]			14			6			8							
Etiology TOAST:[d]			11			7			4							
Etiology TOAST:[e]			7			4			3							
Etiology TOAST:[f]			5			1			4							
Etiology TOAST:[g]			5			3			2							
Etiology TOAST:[h]			3			2			1							
Etiology TOAST:[i]			1			0			1	6.38	8		0.60	0.30		
Duration of hospitalization	5.68	2.67	74	5.84	2.32	34	5.33	3.10	40	836.00		-1.69	0.09	836.00	-0.17	1.75
NIHSS onset	0.57	0.71	77	1.10	1.00	33	0.32	0.48	44	4.74	1,20		0.04			
NIHSS 3m	0.00	0.00	77	0.21	0.42	31	0.00	0.00	46	11.48	1,21.42		0.00			
NIHSS interaction										2.14	1,21.42		0.16			
Stroke localization																
**Lesion site:No lesion**			**14**			**7**			**7**							

**Lesion site:Infratentorial**			**13**			**2**			**11**				
Lesion site:Supratentorial			59			28			31	4.80	2	0.09	0.24
Psychological outcomes													
MoCA onset	26.67	1.58	86	26.43	1.53	37	26.84	1.70	49	0.82	1,39.41	0.37	
MoCA 3m	26.26	1.21	77	26.05	1.29	34	26.44	1.28	43	2.03	1,39.97	0.16	
MoCA interaction										0.25	1,39.97	0.62	
**HAD onset**	**10.06**	**3.03**	**82**	**10.32**	**3.54**	**36**	**9.89**	**2.71**	**46**	**8.38**	**1,38.27**	**0.01**	
HAD 3m	10.65	3.94	71	14.27	4.18	34	7.52	2.86	37	2.31	1,33.1	0.14	
**HAD interaction**										**9.08**	**1,33.1**	**0.00**	
**FIS Cognitive onset**	**26.36**	**13.22**	**72**	**29.75**	**15.97**	**32**	**24.17**	**11.63**	**40**	**16.72**	**1,31.06**	**0.00**	
**FIS cognitive 3m**	**42.43**	**19.04**	**68**	**65.24**	**15.93**	**32**	**24.89**	**12.64**	**36**	**17.42**	**1,31.47**	**0.00**	
**FIS cognitive interaction**										**13.87**	**1,31.47**	**0.00**	
**FIS Physical onset**	**30.74**	**13.25**	**72**	**35.25**	**17.84**	**32**	**28.02**	**10.05**	**40**	**9.65**	**1,33.11**	**0.00**	
**FIS Physical 3m**	**46.86**	**15.09**	**68**	**60.53**	**8.24**	**32**	**31.36**	**13.40**	**36**	**8.52**	**1,33.72**	**0.01**	
**FIS Physical interaction**										**5.28**	**1,33.72**	**0.03**	
**FIS Psychosocial onset**	**27.41**	**9.99**	**72**	**31.06**	**11.64**	**32**	**25.05**	**9.76**	**40**	**21.81**	**1,33.85**	**0.00**	
**FIS Psychosocial 3m**	**40.28**	**16.73**	**68**	**58.03**	**9.66**	**32**	**23.07**	**12.33**	**36**	**13.85**	**1,33.98**	**0.00**	
**FIS Psychosocial interaction**										**22.71**	**1,33.98**	**0.00**	

Note:

The columns represent count data for Chi-square statistics (X^2), Median/IQR for Wilcox signed-rank tests (Ws), trimmed mean±SD for robust ANOVAs (F), mean±SD for independent-samples t-tests and there associated statistical tests and effect sizes. The lines in bold refer to the significant results; Df=Degrees of Freedom; ES=Effect Size; IQR=Inter Quartile Range; N=Sample size; OR=Odds Ratio; SD=Standard Deviation; Stat=Test Statistic; TMean=20% Trimmed Mean; z=Z-value (Wilcoxon test); [B]=statistics for main effect of Between-subject factor 'Group'; [W]=statistics for main effect of Within-subject factor 'Time'; [I]=statistics for Interaction effect ('Group' x 'Time')

[a] Unknown with incomplete evaluation
[b] Unknown despite complete evaluation
[c] Small vessel disease
[d] Patent Foramen Ovale
[e] Cardiac embolism
[f] Cervical artery dissection
[g] Large artery atherosclerosis
[h] More than one possible etiology
[i] Other determined etiology

5 REGRESSION MODELS

5.1 Binomial Logistic Regression Model - RTpW3m_Percent (Yes/No)

The assumptions of i) linearity of the logit regarding the continuous independent variables, ii) independent errors and iii) homoscedasticity were verified respectively, i) by running the analysis with only the interact term between each predictor and its log transform (see Field 51), and by relying on the ii) Durbin-Watson statistic 52,53 and iii) the tolerance and VIF statistics (i.e. entire predictors, i.e. all participants' data for one variable, were rejected if their VIF > 10 or Tolerance < 0.1; see Field, 2009, p.242).

Table S32: Coefficients

	Estimate	Std. Error	z value	Pr(>|z|)	2.5 %	97.5 %
(Intercept)	1.827	0.835	2.187	0.029	0.321	3.660
Hyperlipidemia_onsetYes	-1.329	0.655	-2.030	0.042	-2.730	-0.117
NIHSS_onset	-0.684	0.248	-2.760	0.006	-1.204	-0.223
HAD_TOTAL_onset	0.001	0.041	0.032	0.974	-0.079	0.085

[1] AIC = 93.674
[2] Null deviance = 98.922
[3] Residual deviance = 85.674

Table S33: Deviance residuals

	Df	Deviance	Resid. Df	Resid. Dev	Pr(>Chi)
NULL			71	98.922	
Hyperlipidemia_onset	1	4.419	70	94.504	0.036
NIHSS_onset	1	8.829	69	85.675	0.003
HAD_TOTAL_onset	1	0.001	68	85.674	0.974

Note:
The difference between the null deviance and the residual deviance shows how our model is doing against the null model (a model with only the intercept). The wider this gap, the better. Analyzing the table we can see the drop in deviance when adding each variable one at a time.Ultimately what you would like to see is a significant drop in deviance and the AIC.

Table S34: Model Fit Measures

Model	Deviance	AIC	X2	df	p
1	85.674	93.674	13.248	3	0.004

Table S35: Pseudo R^2 for logistic regression

	R2
Hosmer and Lemeshow R^2:	0.134
Cox and Snell R^2:	0.168
Nagelkerke R^2:	0.225

Table S36: Odds Ratio

	Odds	2.5 %	97.5 %
(Intercept)	6.218	1.209	31.975
Hyperlipidemia_onsetYes	0.265	0.073	0.955
NIHSS_onset	0.505	0.310	0.820
HAD_TOTAL_onset	1.001	0.923	1.086

Table S37: Residuals statistics

	standardized.residuals	studentized.residuals	cooks.distance	mahalanobis.distance	dfbeta..Intercept.	dfbeta.Hyperlipidemia_onsetYes	dfbeta.NIHSS_onset	dfbeta.HAD_TOTAL_onset	dffit	leverage	covariance.ratios	fitted.values
1	-1.121	-1.115	0.007	1.836	-0.054	-0.036	-0.009	0.005	-0.184	0.033	1.034	0.455
3	-0.472	-0.464	0.002	1.109	0.056	-0.032	-0.028	-0.002	-0.115	0.070	1.129	0.098
4	-1.422	-1.418	0.013	2.664	-0.068	-0.038	0.032	0.002	-0.224	0.030	0.994	0.625
5	1.004	0.993	0.010	1.607	0.127	0.007	-0.021	-0.007	0.224	0.059	1.076	0.622
6	-1.124	-1.119	0.007	1.846	0.061	-0.067	-0.007	-0.004	-0.174	0.029	1.030	0.458
7	-1.232	-1.192	0.067	1.829	0.096	0.119	-0.069	-0.016	-0.558	0.205	1.242	0.453
9	-1.118	-1.114	0.006	1.839	-0.028	-0.043	-0.009	0.003	-0.163	0.026	1.027	0.456
10	1.354	1.333	0.052	2.222	0.061	-0.168	0.076	0.005	0.466	0.129	1.117	0.450
14	-0.861	-0.852	0.006	1.421	-0.019	-0.034	-0.032	0.004	-0.178	0.052	1.081	0.296
15	-0.862	-0.855	0.005	1.427	0.071	-0.058	-0.030	-0.003	-0.161	0.043	1.070	0.299
16	-1.166	-1.142	0.034	1.811	-0.150	0.170	-0.068	0.003	-0.396	0.127	1.139	0.448
17	0.761	0.751	0.005	1.314	0.120	-0.108	-0.005	-0.001	0.166	0.057	1.095	0.761
18	0.988	0.982	0.006	1.602	0.069	0.021	-0.022	-0.003	0.166	0.034	1.050	0.624
19	-0.870	-0.857	0.009	1.419	-0.057	-0.025	-0.033	0.007	-0.220	0.075	1.107	0.295
20	0.773	0.760	0.007	1.317	0.178	-0.125	-0.004	-0.005	0.201	0.079	1.120	0.760
21	-2.046	-2.074	0.083	7.336	-0.276	0.243	0.055	-0.001	-0.414	0.047	0.912	0.864
22	1.279	1.271	0.016	2.175	-0.129	0.093	0.006	0.009	0.261	0.050	1.034	0.460
23	-0.858	-0.851	0.004	1.425	0.035	-0.048	-0.030	0.000	-0.151	0.038	1.065	0.298
24	1.283	1.277	0.014	2.199	0.091	0.034	0.012	-0.008	0.243	0.043	1.026	0.455
25	0.985	0.979	0.006	1.596	-0.019	0.044	-0.024	0.004	0.170	0.036	1.052	0.627
26	-0.859	-0.851	0.005	1.423	-0.001	-0.039	-0.031	0.003	-0.164	0.044	1.072	0.297
27	0.563	0.555	0.003	1.160	0.122	-0.080	-0.014	-0.003	0.131	0.065	1.118	0.862
28	0.777	0.758	0.011	1.307	0.008	-0.082	-0.008	0.008	0.245	0.112	1.161	0.765
29	-1.427	-1.423	0.015	2.675	0.011	-0.059	0.034	-0.004	-0.237	0.033	0.997	0.626
31	0.579	0.559	0.008	1.153	-0.024	-0.044	-0.019	0.008	0.214	0.149	1.227	0.867
33	0.557	0.551	0.002	1.159	0.098	-0.072	-0.015	-0.001	0.115	0.051	1.102	0.863
35	-1.453	-1.447	0.031	2.689	0.109	-0.087	0.038	-0.012	-0.339	0.063	1.025	0.628
36	0.557	0.550	0.002	1.159	0.093	-0.071	-0.015	-0.001	0.113	0.050	1.100	0.863
37	0.991	0.982	0.008	1.594	-0.052	0.053	-0.025	0.006	0.203	0.050	1.067	0.627
38	1.586	1.585	0.024	3.354	-0.064	0.090	0.056	0.000	0.281	0.038	0.979	0.298
39	-0.477	-0.467	0.003	1.110	0.074	-0.037	-0.029	-0.003	-0.127	0.083	1.145	0.099
40	0.985	0.979	0.006	1.596	-0.019	0.044	-0.024	0.004	0.170	0.036	1.052	0.627
41	-1.423	-1.420	0.013	2.671	-0.020	-0.051	0.034	-0.002	-0.223	0.030	0.994	0.626
42	0.565	0.556	0.003	1.160	0.127	-0.081	-0.014	-0.004	0.136	0.069	1.123	0.862
43	0.993	0.983	0.009	1.593	-0.063	0.057	-0.026	0.007	0.216	0.056	1.074	0.628
45	1.586	1.585	0.024	3.354	-0.064	0.090	0.056	0.000	0.281	0.038	0.979	0.298
47	0.984	0.979	0.005	1.600	0.036	0.029	-0.023	-0.001	0.152	0.029	1.044	0.625
49	-2.067	-2.110	0.133	7.235	-0.484	0.303	0.052	0.014	-0.527	0.073	0.933	0.862
50	0.996	0.988	0.008	1.605	0.103	0.012	-0.022	-0.006	0.197	0.047	1.063	0.623
51	0.984	0.978	0.005	1.597	-0.008	0.041	-0.024	0.003	0.163	0.033	1.049	0.626
52	-0.643	-0.635	0.004	1.215	0.057	-0.042	-0.034	-0.001	-0.142	0.058	1.105	0.177
53	0.983	0.978	0.005	1.599	0.025	0.032	-0.023	0.000	0.151	0.029	1.044	0.625
54	-1.138	-1.130	0.012	1.851	0.115	-0.082	-0.006	-0.008	-0.232	0.050	1.050	0.460
55	0.993	0.983	0.009	1.593	-0.063	0.057	-0.026	0.007	0.216	0.056	1.074	0.628
56	-0.650	-0.639	0.004	1.217	0.087	-0.051	-0.034	-0.003	-0.159	0.071	1.120	0.178
57	0.983	0.978	0.005	1.598	0.003	0.038	-0.023	0.002	0.157	0.031	1.046	0.626
58	0.991	0.984	0.006	1.603	0.080	0.018	-0.022	-0.004	0.175	0.038	1.053	0.624
59	0.984	0.979	0.005	1.600	0.036	0.029	-0.023	-0.001	0.152	0.029	1.044	0.625
60	1.591	1.590	0.027	3.363	-0.015	0.077	0.057	-0.004	0.297	0.041	0.982	0.297
62	0.760	0.750	0.005	1.313	0.105	-0.104	-0.005	0.000	0.165	0.056	1.095	0.762
63	-1.422	-1.419	0.013	2.669	-0.036	-0.047	0.033	0.000	-0.220	0.029	0.993	0.625
64	-1.423	-1.420	0.015	2.660	-0.099	-0.030	0.032	0.004	-0.241	0.034	0.999	0.624
65	0.759	0.750	0.005	1.312	0.090	-0.100	-0.005	0.001	0.168	0.058	1.097	0.762
66	-0.863	-0.853	0.007	1.421	-0.028	-0.032	-0.032	0.005	-0.187	0.056	1.086	0.296
68	1.270	1.266	0.008	2.192	0.032	0.049	0.010	-0.003	0.186	0.026	1.010	0.456
69	-1.453	-1.447	0.031	2.689	0.109	-0.087	0.038	-0.012	-0.339	0.063	1.025	0.628
70	0.557	0.551	0.002	1.159	0.098	-0.072	-0.015	-0.001	0.115	0.051	1.102	0.863
71	-0.641	-0.633	0.004	1.214	0.028	-0.034	-0.035	0.001	-0.142	0.058	1.105	0.176
72	0.561	0.550	0.004	1.155	0.018	-0.052	-0.017	0.005	0.151	0.085	1.142	0.866
73	1.268	1.264	0.008	2.191	0.017	0.052	0.010	-0.002	0.178	0.024	1.008	0.456
74	0.786	0.768	0.010	1.318	0.216	-0.138	-0.003	-0.008	0.240	0.106	1.152	0.759
75	1.360	1.339	0.053	2.235	0.192	-0.203	0.079	-0.005	0.469	0.130	1.117	0.447
76	-1.422	-1.418	0.013	2.664	-0.068	-0.038	0.032	0.002	-0.224	0.030	0.994	0.625
77	-2.052	-2.087	0.103	7.268	-0.410	0.281	0.053	0.009	-0.462	0.058	0.921	0.862
80	-0.859	-0.851	0.005	1.423	-0.001	-0.039	-0.031	0.003	-0.164	0.044	1.072	0.297
81	0.986	0.981	0.005	1.602	0.058	0.024	-0.022	-0.002	0.159	0.032	1.047	0.624
85	0.988	0.980	0.007	1.594	-0.041	0.050	-0.025	0.005	0.190	0.045	1.061	0.627
91	1.000	0.990	0.009	1.606	0.115	0.009	-0.022	-0.007	0.210	0.053	1.069	0.623
92	1.272	1.268	0.009	2.194	0.046	0.045	0.010	-0.004	0.197	0.029	1.013	0.456
93	-1.430	-1.426	0.016	2.678	0.027	-0.064	0.035	-0.005	-0.249	0.036	1.000	0.627
94	-0.858	-0.851	0.005	1.423	0.008	-0.041	-0.031	0.002	-0.158	0.041	1.069	0.297
95	-1.425	-1.421	0.017	2.658	-0.116	-0.026	0.032	0.006	-0.253	0.038	1.002	0.624

Table S38: Assumption of linearity of the logit (for continuous predictors)

	Estimate	Std. Error	z value	Pr(>|z|)
(Intercept)	-26.230	3827.021	-0.007	0.995
Hyperlipidemia_onsetYes	-19.672	2320.244	-0.008	0.993
NIHSS_onset	43.557	6147.260	0.007	0.994
HAD_TOTAL_onset	1.010	1.071	0.943	0.346
LogNIHSS_onsetInt	-32.331	4434.310	-0.007	0.994
LogHAD_TOTAL_onset_onsetInt	-0.308	0.313	-0.982	0.326

Table S39: Durbin-Watson test (Assumption of independence)

Autocorrelation	DWStatistic	p
-0.087	2.138	0.57

Note:
<1 or >3 is cause for concern. If close to 2 = assumption met

Table S40: Colinearity Diagnostics

	VIF	Tolerance
Hyperlipidemia_onset	1.088	0.919
NIHSS_onset	1.038	0.964
HAD_TOTAL_onset	1.060	0.943

Table S41: Classification Table

	False	True	% Change
NoRTpW	19	13	59.375
RTpW	10	30	75.000

Table S42: Predictive Measures

Accuracy	Specificity	Sensivity	AUC	Cutoff
0.681	0.594	0.75	0.729	0.5

Figure S27: ROC Curve

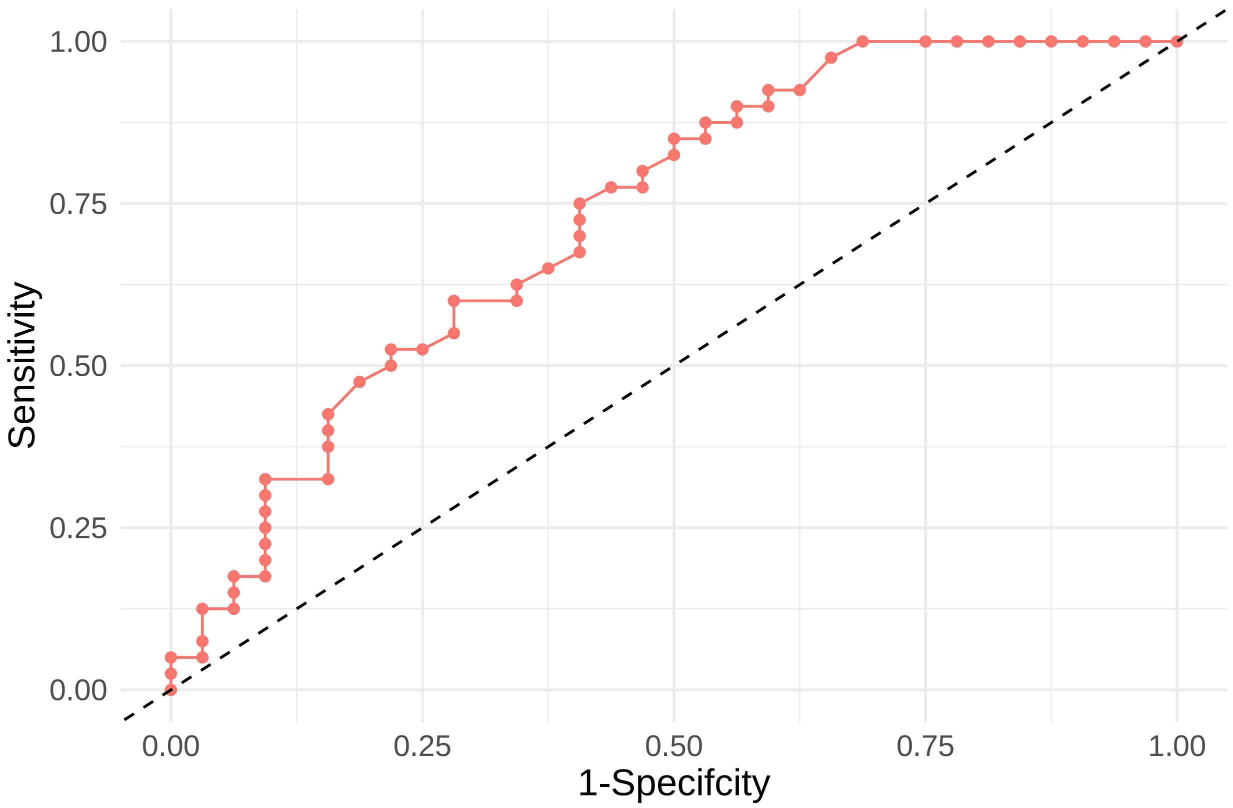

Note: ROC Curve for the binary logistic regression model

5.2 Negative Binomial Regression Model - RTpWMonths

Since the distribution of our dependent variable was right-skewed (see Figure 2), we computed a negative binomial regression model with log link function (Beaujean and Grant, 2016).

To verify the model's assumptions, we i) ran a likelihood ratio test contrasting our negative binomial regression model against a Poisson regression model (i.e. p<.001 favoring the former) and further ii) interpreted the dispersion parameter of the distribution theta ( ) in the range between 0 and 4.

Table S43: Mean and SD for the RTpWMonths DV

Mean	SD
1.986	2.447

Table S44: Coefficients

	Estimate	Std. Error	z value	Pr(>|z|)	2.5 %	97.5 %
(Intercept)	-0.461	0.454	-1.016	0.310	-1.429	0.462
Hyperlipidemia_onsetYes	0.737	0.353	2.091	0.037	0.022	1.477
NIHSS_onset	0.220	0.120	1.831	0.067	-0.025	0.476
HAD_TOTAL_onset	0.040	0.021	1.950	0.051	0.001	0.080

[1] AIC = 235.003

[2] Null deviance: = 73.173

[3] Residual deviance: = 64.655

[4] Theta: = 1.470

[5] Theta SEr: = 0.515

[6] 2x log-likelihood: = -225.003

Table S45: Pseudo R^2 values

	R2
McFadden	0.201
Cox and Snell (ML)	0.617
Nagelkerke (Cragg and Uhler)	0.623

Table S46: Omnibus test

Model	theta	Resid. df	2 x log-lik.	Test	df	LR stat.	Pr(Chi)
1	1.096	73	-281.699				
Hyperlipidemia_onset + NIHSS_onset + HAD_TOTAL_onset	1.470	55	-225.003	1 vs 2	18	56.696	0

Table S47: Incidence rate ratios

	exp(Est.)	2.5%	97.5%	z val.	p
(Intercept)	0.631	0.259	1.534	-1.016	0.310
Hyperlipidemia_onsetYes	2.090	1.047	4.170	2.091	0.037
NIHSS_onset	1.246	0.985	1.577	1.831	0.067
HAD_TOTAL_onset	1.041	1.000	1.084	1.950	0.051

Table S48: MODEL ASSUMPTION: Likelihood ratio test Negative Binomial Reg > Poisson Reg models

Pvalue
0

Table S49: Residuals statistics

	standardized.residuals	studentized.residuals	cooks.distance	mahalanobis.distance	dfbeta..Intercept.	dfbeta.Hyperlipidemia_onsetYes	dfbeta.NIHSS_onset	dfbeta.HAD_TOTAL_onset	dffit	leverage	covariance.ratios	fitted.values
1	0.414	0.380	0.002	0.495	0.013	0.006	0.001	-0.001	0.069	0.032	1.100	2.007
3	-1.129	-0.991	0.048	0.682	0.130	-0.075	-0.061	-0.004	-0.546	0.215	1.266	6.282
5	0.359	0.330	0.002	0.458	0.023	0.001	-0.004	-0.001	0.076	0.051	1.125	1.372
7	1.564	1.598	0.307	1.934	-0.045	-0.086	0.045	0.009	0.727	0.199	1.152	3.749
10	-0.222	-0.202	0.002	0.203	-0.009	0.016	-0.006	0.000	-0.076	0.122	1.222	2.511
14	0.563	0.521	0.006	0.665	0.013	0.009	0.009	-0.001	0.126	0.056	1.118	2.403
16	0.052	0.048	0.000	0.054	0.004	-0.004	0.001	0.000	0.017	0.118	1.219	1.897
17	-1.397	-1.271	0.012	1.000	-0.133	0.111	0.003	0.002	-0.331	0.061	1.014	1.271
18	-1.523	-1.401	0.007	1.000	-0.057	-0.019	0.017	0.002	-0.271	0.035	0.964	1.676
19	1.063	1.006	0.040	1.443	0.051	0.007	0.017	-0.004	0.282	0.077	1.086	2.047
20	0.032	0.030	0.000	0.041	0.004	-0.003	0.000	0.000	0.009	0.077	1.166	0.960
22	-0.487	-0.443	0.003	0.408	0.027	-0.023	0.000	-0.002	-0.106	0.054	1.121	3.379
24	-1.580	-1.453	0.009	1.000	-0.066	-0.015	-0.003	0.004	-0.299	0.040	0.957	1.852
25	-0.668	-0.608	0.003	0.567	0.007	-0.021	0.009	-0.001	-0.126	0.041	1.091	2.310
26	-1.775	-1.635	0.013	1.000	-0.018	-0.036	-0.029	0.003	-0.377	0.049	0.927	2.603
27	-1.141	-1.029	0.011	1.000	-0.150	0.096	0.013	0.004	-0.308	0.079	1.077	0.740
28	-0.145	-0.132	0.001	0.138	-0.002	0.008	0.001	-0.001	-0.050	0.123	1.226	2.319
31	-0.307	-0.276	0.007	0.251	0.011	0.014	0.007	-0.003	-0.169	0.266	1.458	2.669
33	-1.230	-1.114	0.011	1.000	-0.140	0.099	0.016	0.003	-0.308	0.068	1.051	0.905
36	-1.249	-1.132	0.011	1.000	-0.137	0.099	0.016	0.002	-0.310	0.067	1.046	0.942
37	-0.769	-0.699	0.006	0.616	0.024	-0.030	0.011	-0.002	-0.172	0.056	1.099	2.605
38	-0.393	-0.358	0.001	0.346	0.006	-0.012	-0.006	0.000	-0.077	0.044	1.115	3.056
40	0.269	0.247	0.001	0.299	-0.003	0.008	-0.003	0.000	0.051	0.041	1.117	2.310
41	1.550	1.471	0.045	2.418	0.012	0.037	-0.019	0.001	0.268	0.033	0.957	2.048
42	-1.124	-1.012	0.012	1.000	-0.151	0.096	0.013	0.004	-0.309	0.082	1.083	0.711
43	-0.287	-0.261	0.001	0.262	0.011	-0.012	0.004	-0.001	-0.068	0.063	1.142	2.711
45	-0.890	-0.811	0.005	0.673	0.015	-0.027	-0.014	0.000	-0.175	0.044	1.071	3.056
47	-1.585	-1.462	0.007	1.000	-0.032	-0.030	0.019	0.001	-0.269	0.032	0.949	1.890
50	-1.465	-1.343	0.009	1.000	-0.079	-0.008	0.016	0.004	-0.290	0.043	0.983	1.486
51	0.648	0.599	0.006	0.803	-0.003	0.019	-0.008	0.001	0.117	0.037	1.089	2.219
52	-1.190	-1.072	0.020	0.767	0.068	-0.054	-0.039	-0.001	-0.369	0.101	1.096	4.295
53	-0.537	-0.490	0.002	0.492	-0.008	-0.012	0.007	0.000	-0.090	0.032	1.092	1.968
54	1.084	1.019	0.029	1.368	-0.060	0.051	0.001	0.004	0.239	0.054	1.057	3.379
55	-1.811	-1.661	0.017	1.000	0.068	-0.077	0.025	-0.006	-0.440	0.063	0.932	2.711
56	-0.295	-0.267	0.003	0.238	0.029	-0.018	-0.010	-0.001	-0.104	0.130	1.230	5.247
57	-0.602	-0.549	0.002	0.531	-0.001	-0.016	0.008	0.000	-0.105	0.035	1.090	2.132
58	-1.503	-1.381	0.008	1.000	-0.064	-0.015	0.017	0.003	-0.276	0.037	0.970	1.610
59	-1.585	-1.462	0.007	1.000	-0.032	-0.030	0.019	0.001	-0.269	0.032	0.949	1.890
60	0.104	0.095	0.000	0.107	0.000	0.002	0.002	0.000	0.021	0.046	1.128	2.710
63	2.612	2.617	0.189	5.098	0.037	0.056	-0.032	0.000	0.461	0.032	0.712	1.968
64	1.831	1.762	0.075	3.176	0.068	0.022	-0.021	-0.003	0.328	0.035	0.901	1.676
65	0.739	0.688	0.013	1.010	0.056	-0.055	-0.002	0.000	0.174	0.062	1.109	1.492
66	0.272	0.250	0.001	0.300	0.008	0.004	0.004	-0.001	0.063	0.060	1.140	2.308
68	0.330	0.302	0.001	0.380	0.006	0.006	0.001	0.000	0.050	0.027	1.099	2.174
69	0.385	0.355	0.003	0.417	-0.017	0.017	-0.005	0.002	0.097	0.071	1.147	2.822
70	-1.230	-1.114	0.011	1.000	-0.140	0.099	0.016	0.003	-0.308	0.068	1.051	0.905
71	-0.164	-0.149	0.001	0.146	0.003	-0.005	-0.005	0.000	-0.049	0.096	1.189	3.515
72	-0.520	-0.468	0.008	0.463	-0.014	0.031	0.009	-0.002	-0.185	0.131	1.218	1.861
73	-1.677	-1.553	0.006	1.000	-0.020	-0.035	-0.003	0.001	-0.254	0.026	0.924	2.263
74	0.161	0.148	0.001	0.222	0.023	-0.015	0.000	-0.001	0.047	0.092	1.184	0.818
75	0.541	0.504	0.013	0.646	0.048	-0.047	0.014	-0.001	0.182	0.120	1.200	1.822
76	-0.474	-0.432	0.001	0.449	-0.012	-0.008	0.006	0.000	-0.079	0.032	1.097	1.816
77	0.879	0.826	0.025	1.493	0.109	-0.073	-0.011	-0.003	0.228	0.074	1.107	0.802
81	-1.543	-1.421	0.007	1.000	-0.049	-0.022	0.018	0.002	-0.268	0.034	0.958	1.745
85	-0.210	-0.192	0.001	0.201	0.005	-0.008	0.003	-0.001	-0.044	0.050	1.130	2.503
91	0.780	0.725	0.011	1.101	0.046	0.003	-0.008	-0.002	0.159	0.047	1.087	1.428
92	-1.637	-1.513	0.007	1.000	-0.040	-0.026	-0.003	0.003	-0.266	0.029	0.936	2.089
93	-1.701	-1.569	0.010	1.000	0.018	-0.053	0.022	-0.003	-0.328	0.041	0.934	2.310
95	0.648	0.599	0.006	0.863	0.028	0.006	-0.007	-0.001	0.117	0.037	1.089	1.610

Table S50: Durbin-Watson test (Assumption of independence)

Autocorrelation	DWStatistic	p
0.253	1.485	0.044

Note:

<1 or >3 is cause for concern. If close to 2 = assumption met

Table S51: Colinearity Diagnostics

	VIF	Tolerance
Hyperlipidemia_onset	1.131	0.884
NIHSS_onset	1.012	0.988
HAD_TOTAL_onset	1.125	0.889

Figure S28: Number of months needed to RTpW

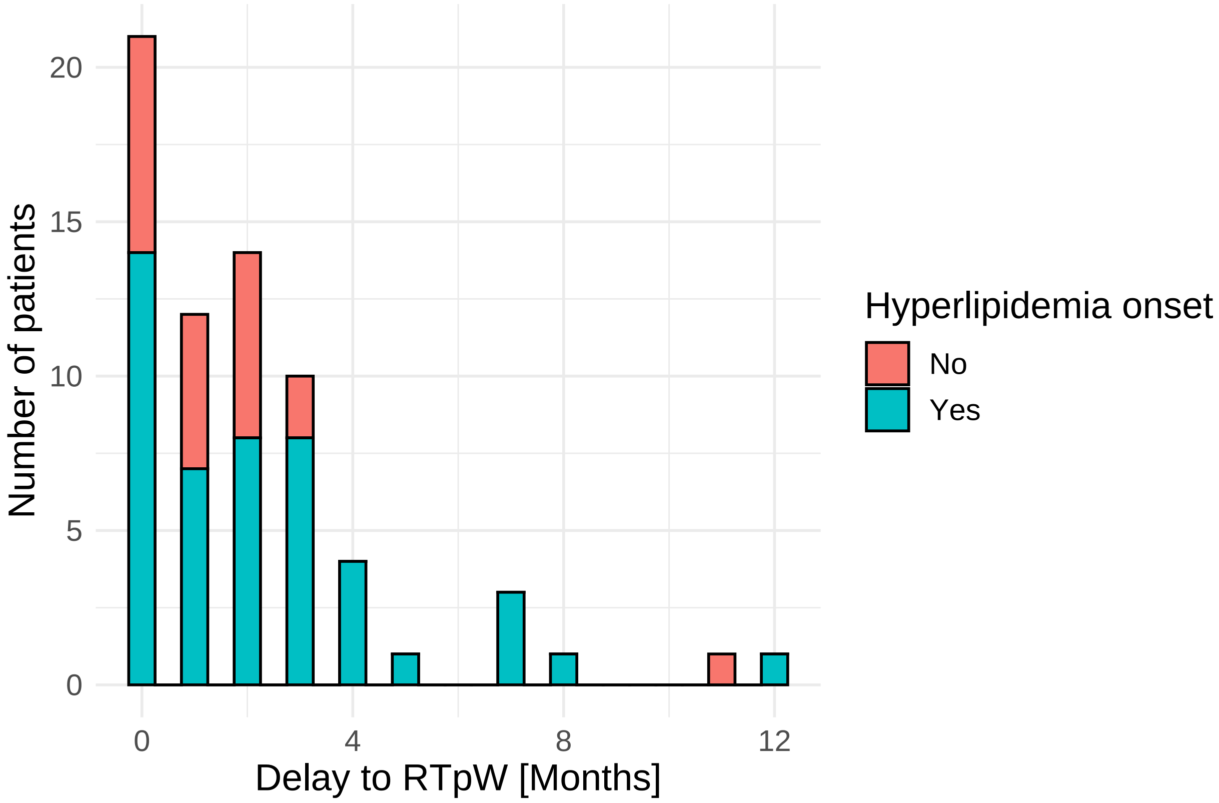

Note: Histogram of the number of months needed to RTpW by Hyperlipidemia

Figure S29: Assumption of heteroscedasticity

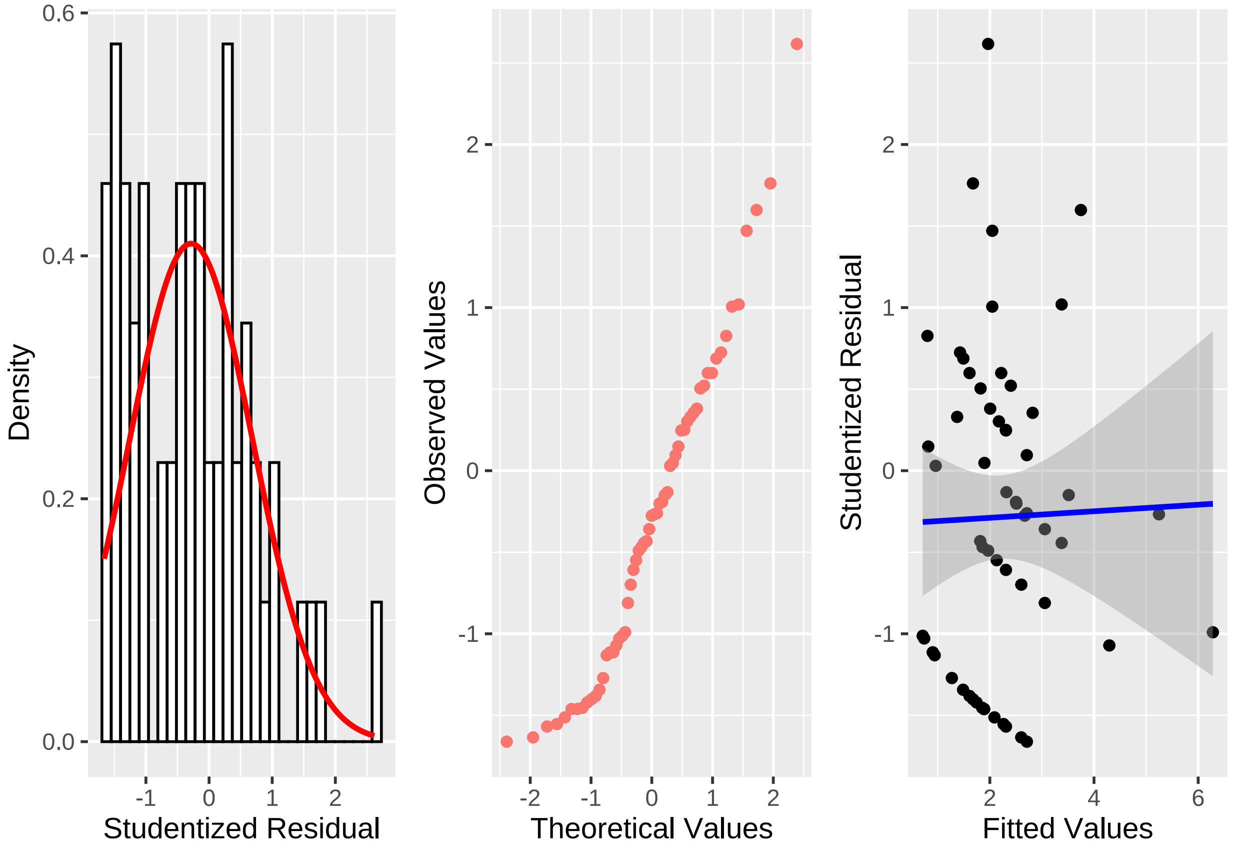

Table S52: Breusch-Pagan test for heteroskedasticity

	BP	Df	p
BP	1.598	3	0.66

Table S53: Predicted probabilities

Hyperlipidemia_onset	HAD_TOTAL_onset	NIHSS_onset	PredictedRTpWMonths
No	10.797	0.859	1.175
Yes	10.797	0.859	2.455

6 CAUSAL MEDIATION MODEL

To further assess whether NIHSS could mediate the predictive effect of hyperlipidemia over the likelihood to RTpW at 3 months (RTpW3m_Percent (Yes/No)), we ran a mediation analysis using the mediation R package.

To verify the model's assumptions, we ran a sensitivity analysis using the medsens function.

Table S54: Total effect (IV –> DV)

	Estimate	Std. Error	z value	Pr(>|z|)
(Intercept)	1.099	0.516	2.127	0.033
Hyperlipidemia_onsetYes	-1.176	0.586	-2.005	0.045

Table S55: Mediator effect (IV –> mediator)

	Estimate	Std. Error	t value	Pr(>|t|)
(Intercept)	0.900	0.258	3.483	0.001
Hyperlipidemia_onsetYes	0.023	0.304	0.076	0.940

Table S56: Direct effect (IV + mediator –> DV)

	Estimate	Std. Error	z value	Pr(>|z|)
(Intercept)	1.845	0.634	2.909	0.004
Hyperlipidemia_onsetYes	-1.334	0.639	-2.088	0.037
NIHSS_onset	-0.684	0.248	-2.760	0.006

Table S57: Mediation analysis

	Estimate	2.5CI	97.5CI	P.value
ACME (control)	-0.003	-0.085	0.071	0.920
ACME (treated)	-0.003	-0.097	0.098	0.920
ADE (control)	-0.275	-0.505	-0.033	0.028
ADE (treated)	-0.275	-0.517	-0.035	0.028
Total Effect	-0.278	-0.514	-0.020	0.036
Prop. Mediated (control)	0.010	-0.774	0.478	0.916
Prop. Mediated (treated)	0.012	-0.784	0.490	0.916
ACME (average)	-0.003	-0.092	0.083	0.920
ADE (average)	-0.275	-0.509	-0.034	0.028
Prop. Mediated (average)	0.011	-0.758	0.484	0.916

[1] ACME = Average Causal Mediation Effects, i.e. indirect effect of IV on DV going through the mediator.

[2] ADE = Average Direct Effects, i.e. direct effect of IV on the DV

[3] Total Effect = direct + indirect effects on the DV

[4] Prop. Mediated = proportion of IV effect on the DV going through the mediator, i.e. ACME/total effect

```
## [1] 1
```

```
## [1] "Rho at which ACME for Control Group = 0: -0.800"
```

Table S58: Sensitivity analysis

Rho	ACME(C)	2.5CI(C)	97.5CI(C)	ACME(T)	2.5CI(T)	97.5CI(T)	R^2_M*R^2_Y*	R^2_M~R^2_Y~
-0.9	-0.006	-0.285	0.187	0.019	-0.276	0.304	0.81	0.599
-0.8	0.000	-0.164	0.123	0.012	-0.170	0.189	0.64	0.473
-0.7	0.001	-0.105	0.083	0.007	-0.114	0.127	0.49	0.362
-0.6	0.001	-0.069	0.059	0.005	-0.075	0.085	0.36	0.266
-0.5	0.001	-0.043	0.038	0.003	-0.050	0.052	0.25	0.185
-0.4	0.000	-0.023	0.022	0.001	-0.027	0.033	0.16	0.118
-0.3	0.000	-0.015	0.017	-0.001	-0.023	0.022	0.09	0.067
-0.2	-0.001	-0.025	0.026	-0.002	-0.040	0.031	0.04	0.030
-0.1	-0.002	-0.040	0.038	-0.003	-0.056	0.049	0.01	0.007
0.0	-0.003	-0.054	0.050	-0.004	-0.073	0.065	0.00	0.000
0.1	-0.004	-0.068	0.062	-0.005	-0.094	0.082	0.01	0.007
0.2	-0.005	-0.083	0.076	-0.007	-0.115	0.099	0.04	0.030
0.3	-0.007	-0.100	0.091	-0.008	-0.135	0.119	0.09	0.067
0.4	-0.008	-0.123	0.106	-0.009	-0.155	0.139	0.16	0.118
0.5	-0.011	-0.150	0.120	-0.011	-0.186	0.161	0.25	0.185
0.6	-0.014	-0.179	0.138	-0.012	-0.216	0.190	0.36	0.266
0.7	-0.018	-0.226	0.162	-0.015	-0.255	0.226	0.49	0.362
0.8	-0.026	-0.297	0.191	-0.018	-0.310	0.277	0.64	0.473
0.9	-0.044	-0.445	0.244	-0.023	-0.394	0.365	0.81	0.599

Note:
C = Control condition; T = Treatment condition

```
## [1] "R^2_M*R^2_Y* at which ACME for Control Group = 0: 0.640"
## [1] "R^2_M~R^2_Y~ at which ACME for Control Group = 0: 0.473"
## [1] "Rho at which ACME for Treatment Group = 0: -0.300"
## [1] "R^2_M*R^2_Y* at which ACME for Treatment Group = 0: 0.090"
## [1] "R^2_M~R^2_Y~ at which ACME for Treatment Group = 0: 0.067"
```

Figure S30: Sensitivity analysis

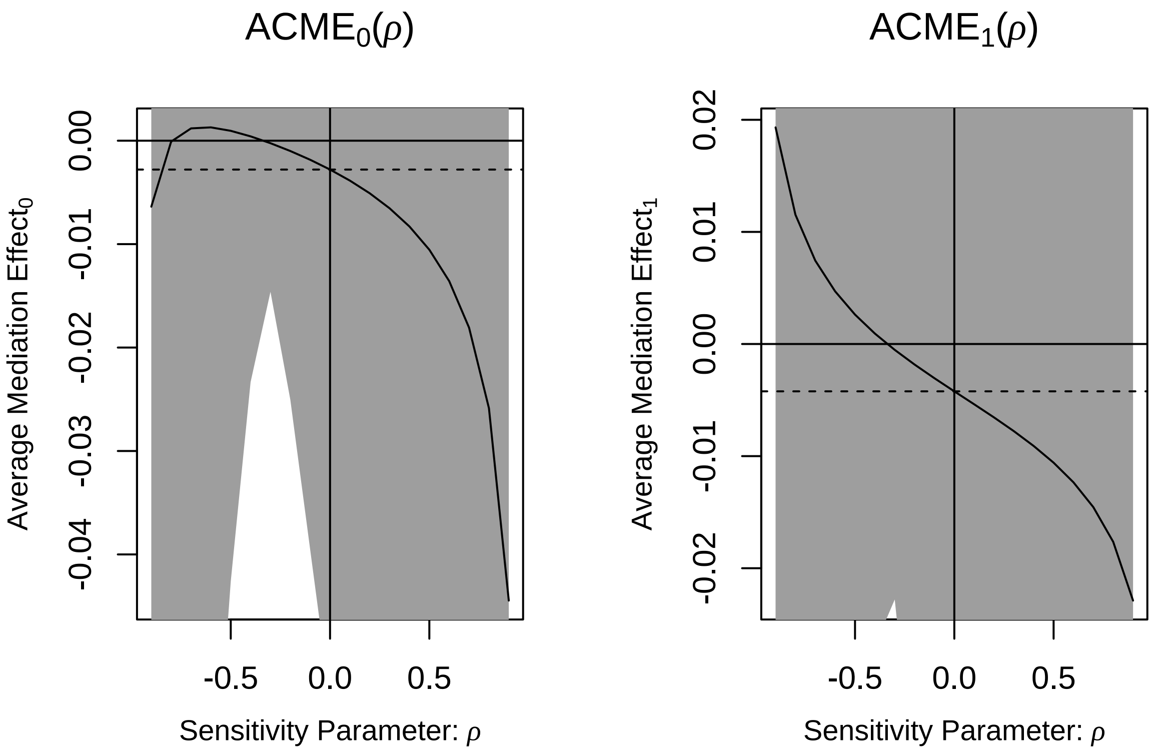

Supplement: Supplementary file 1 [file jpm-12-01109-s001.zip › jpm-1785932-SI.pdf]
